# Supplementary material for: Effect of Trimethine Cyanine Dye- and Folate-Conjugation on the In Vitro Biological Activity of Proapoptotic Peptides
Source: Biomolecules. 2022 May 20;12(5):725. doi: 10.3390/biom12050725 (PMC9138991; doi:10.3390/biom12050725)
Supplement: Supplementary file 1 [file biomolecules-12-00725-s001.zip › biomolecules-1694115-supplementary.pdf]

Table of Contents

1. Experimental Section 2

1.1 Determination of Rink Amide resin loading 2

1.2 Synthesis of cyanine dye scaffold **1** 2

1.3 SPPS of compounds **2**-**4** 2

1.4 SPPS of compounds **5**-**7** 2

1.5 SPPS of compounds **8** and **9** 2

1.6 SPPS of compound **10** 3

1.7 SPPS of compounds **11**-**13** 3

2. Supplementary Figures 4

3. ^{1}H-NMR and high-resolution mass spectrum of **1** 22

4. Table S1. Calculated and found mass to charge ratio for compounds **1**–**13** by mass spectroscopy. 23

4.1 High-resolution mass spectra for compounds **2**-**11** and **13** 23

5. References 25

1. Experimental Section

1.1 Determination of Rink Amide resin loading

10 mg of resin was weighed into an Eppendorf tube, to which 800 µL of DMF was added. After allowing the resin to swell for 15 min, 200 µL of piperidine was added to the suspension; at this point the tube was vortexed and allowed to stand for 15 min at room temperature. Next, the tube was centrifuged and 100 µL of the supernatant transferred to a 1 cm path-length cuvette and diluted with 900 µL of DMF. The absorbance at 301 nm was measured versus a blank, which was prepared with the same procedure but without the use of the resin. The concentration, c, of dibenzofulvene-piperidine adduct released from the resin was determined by applying the Lambert-Beer law [A= ε c l], where A is the absorbance value, l is the path length (1 cm) and ε is the extinction coefficient of the dibenzofulvene-piperidine adduct (7800 mL/mmol*cm, λ = 301 nm) [22]. Average values resulted from three independent experiments. Finally, the loading, L, was calculated using the formula:

L = c x V x d / m

Where V is the volume of solution in the cuvette, d is the dilution coefficient, and m is the mass of the weighted resin. In the described procedure, V = 1 mL, d = 10 and m = 10 mg.

1.2 Synthesis of cyanine dye scaffold **1**

Compound 1 was prepared using the literature procedure [1]. (See section 3 for characterization data)

1.3 SPPS of compounds **2-4**

The peptide chain was synthesized by SPPS on Rink Amide MBHA resin. Each amino acid coupling step was carried out with Fmoc-protected amino acid (2 eq), DIC (4 eq), ethyl cyanohydroxyiminoacetate (2 eq) in DMF at 75 °C (155 W) for 0.25 min and at 90 °C (30 W) for further 2 min. For the last lysine, the coupling was carried out at 75 °C (155 W) for 0.5 min and at 90 °C (30 W) for further 4 min. After each coupling step, the respective Fmoc protecting group was removed by 10% Piperazine (w/v) in ethanol:NMP (1:9). After the final Fmoc deprotection, cleavage was performed in TFA/H2O/TIS 95:2.5:2.5 solution for 2 h. The resin was removed by filtration and the filtrate was concentrated by N_2 flow. The crude product was precipitated with cold diethyl ether, centrifuged (4,000 g for 10 min) and the supernatant was discarded. The precipitate was dissolved in water, filtrated through a 0.22 µm syringe filter and purified by RP-HPLC. Lyophilization of the pure product fractions afforded the desired compound as white powder, which was characterized by LC-MS (Figure S9-S11). Yield 45-35%

1.4 SPPS of compounds **5-7**

The peptide chain was synthesized by SPPS on Rink Amide MBHA resin. Each amino acid coupling step was carried out with Fmoc-protected amino acid (2 eq), DIC (4 eq), ethyl cyanohydroxyiminoacetate (2 eq) in DMF at 75 °C (155 W) for 0.25 min and at 90 °C (30 W) for further 2 min. For the last lysine, the coupling was carried out at 75 °C (155 W) for 0.5 min and at 90 °C (30 W) for a further 4 min. After each coupling step, the respective Fmoc protecting group was removed by 10% Piperazine (w/v) in ethanol:NMP (1:9). After the final Fmoc deprotection, the cyanine dye scaffold was manually coupled using 3 eq of compound 1, 6 eq of DIC, 3 eq of ethyl cyanohydroxyiminoacetate for 16 h at room temperature. Cleavage was performed in TFA/H2O/TIS 95:2.5:2.5 solution for 2 h. The resin was removed by filtration and the filtrate was concentrated by N_2 flow. The crude product was precipitated with cold diethyl ether, centrifuged (4,000 g for 10 min) and the supernatant was discarded. The precipitate was dissolved in water, filtrated through a 0.22 µm syringe filter and purified by RP-HPLC. Lyophilization of the pure product fractions afforded the desired compound as red powder which were characterized by LC-MS (Figure S12-S14). Yield 33-27%

1.5 SPPS of compounds **8** and **9**

The peptide chain was synthesized by SPPS on Rink Amide MBHA resin. Each amino acid coupling step was carried out with Fmoc-protected amino acid (2 eq), DIC (4 eq), ethyl cyanohydroxyiminoacetate (2 eq) in DMF at 75 °C (155 W) for 0.25 min and at 90 °C (30 W) for further 2 min. For the penultimate lysine, the coupling was carried out using Fmoc-Lys(Boc)-OH (2 eq), DIC (4 eq), ethyl cyanohydroxyiminoacetate (2 eq) at 75 °C (155 W) for 0.5 min and at 90 °C (30 W) for further 4 min. For the last lysine, the coupling was carried out using Fmoc-Lys(Mtt)-OH (3 eq), DIC (6 eq), ethyl cyanohydroxyiminoacetate (3 eq) at 75 °C (155 W) for 1 min and at 90 °C (30 W) for further 7 min. After each coupling step, the respective Fmoc protecting group was removed by 10% piperazine (w/v) in ethanol:NMP (1:9). After the final Fmoc deprotection, the peptidyl resin was removed from the automated synthesizer and the trimethine cyanine dye

was manually coupled using 3 eq of compound 1, 6 eq of DIC, 3 eq of ethyl cyanohydroxyiminoacetate, 16 h. For the Mtt group cleavage, the resin was treated with 1% TFA (v/v) in DCM for 2 min and then the solution filtrated off. The procedure was repeated 12 times. Then, glutamic acid was coupled using 4 eq of Fmoc-Glu(OtBu)-OH, 4 eq of HBTU, 4 eq of HOBt and 8 eq of DIPEA. Deprotection of the Fmoc protecting group was achieved by treatment with 5 mL of 20% (v/v) piperidine in DMF, for 10 min for 2 times. For the pteroic acid coupling, 2 eq of pteroic acid were suspended in 10 mL of DMSO and were heated at 50 °C for 1 h. 8 eq of DIPEA and 2 eq of Pybop were added to the suspension which was then reacted with the peptidyl-resin for 16 h at 40 °C. The pteroic acid coupling was then repeated for further 16 h at 40 °C. Cleavage from the resin was performed in TFA/H2O/TIS 95:2.5:2.5 solution for 2 h. The resin was removed by filtration and the filtrate concentrated by N_2 flow. The crude product was precipitated with cold diethyl ether, centrifuged (4,000 g for 10 min) and the supernatant discarded. The precipitate was dissolved in 2 mL of CH_3CN + 0.1% TFA, diluted with 13 mL of H2O + 0.1% TFA, filtrated through a 0.22 µm syringe filter and purified by RP-HPLC. Lyophilization of the pure product fractions afforded the desired compound as a red powder, which was characterized by LC-MS (Figure S15-S16). Yield 7-5%

1.6 SPPS of compound **10**

The peptide chain was synthesized by SPPS on Rink Amide MBHA resin. Each amino acid coupling step was carried out with Fmoc-protected amino acid (2 eq), DIC (4 eq), ethyl cyanohydroxyiminoacetate (2 eq) in DMF at 75 °C (155 W) for 0.25 min and at 90 °C (30 W) for further 2 min. For the penultimate lysine, the coupling was carried out using Fmoc-Lys(Boc)-OH (2 eq), DIC (4 eq), ethyl cyanohydroxyiminoacetate (2 eq) at 75 °C (155 W) for 0.5 min and at 90 °C (30 W) for further 4 min. For the last lysine, the coupling was carried out using Fmoc-Lys(ivDde)-OH (3 eq), DIC (6 eq), ethyl cyanohydroxyiminoacetate (3 eq) at 75 °C (155 W) for 1 min and at 90 °C (30 W) for further 7 min. After each coupling step, the respective Fmoc protecting group was removed by 10% piperazine (w/v) in ethanol:NMP (1:9). After the final Fmoc deprotection, the peptidyl resin was removed from the automated synthesizer and the trimethine cyanine dye was manually coupled using 3 eq of compound 1, 6 eq of DIC, 3 eq of ethyl cyanohydroxyiminoacetate, 16 h. For the ivDde group cleavage, the resin was treated with 4% hydrazine hydrate (v/v) in DMF for 45 min and then the solution filtrated off. The procedure was repeated twice. Then, glutamic acid was coupled using 4 eq of Fmoc-Glu(OtBu)-OH, 4 eq of HBTU, 4 eq of HOBt and 8 eq of DIPEA. Deprotection of the Fmoc protecting group was achieved by treatment with 5 mL of 20% (v/v) piperidine in DMF, for 10 min for 2 times. For the pteroic acid coupling, 2 eq of pteroic acid were suspended in 10 mL of DMSO and heated at 50 °C for 1 h. 8 eq of DIPEA and 2 eq of Pybop were added to the suspension, which was then reacted with the peptidyl-resin for 16 h at 40 °C. The pteroic acid coupling was then repeated for further 16 h at 40 °C. Cleavage from the resin was performed in TFA/H2O/TIS 95:2.5:2.5 solution for 2 h. The resin was removed by filtration and the filtrate concentrated by N2 flow. The crude product was precipitated with cold diethyl ether, centrifuged (4,000 g for 10 min) and the supernatant was discarded. The precipitate was dissolved in 2 mL of CH3CN + 0.1% TFA, diluted with 13 mL of H2O + 0.1% TFA, filtrated through a 0.22 µm syringe filter and purified by RP-HPLC. Lyophilization of the pure product fractions afforded the desired compound as a red powder, which was characterized by LC-MS (Figure S17). Yield 10%

1.7 SPPS of compounds **11-13**

The peptide chain was synthesized by SPPS on Rink Amide MBHA resin. Each amino acid coupling step was carried out with Fmoc-protected amino acid (2 eq), DIC (4 eq), ethyl cyanohydroxyiminoacetate (2 eq) in DMF at 75 °C (155 W) for 0.25 min and at 90 °C (30 W) for further 2 min. For the penultimate lysine, the coupling was carried out using Fmoc-Lys(Boc)-OH (2 eq), DIC (4 eq), ethyl cyanohydroxyiminoacetate (2 eq) at 75 °C (155 W) for 0.5 min and at 90 °C (30 W) for further 4 min. For the last lysine, the coupling was carried out using Fmoc-Lys(Mtt)-OH (3 eq), DIC (6 eq), ethyl cyanohydroxyiminoacetate (3 eq) at 75 °C (155 W) for 1 min and at 90 °C (30 W) for further 7 min. After each coupling step, the respective Fmoc protecting group was removed by 10% Piperazine (w/v) in ethanol:NMP (1:9). After the final Fmoc deprotection, the peptidyl-resin was removed from the automated synthesizer and the N-terminal amine capped using acetic anhydride (50 eq) and pyridine (50 eq) in 8 mL of DMF, for 30 min for 2 times. For the Mtt group cleavage, the resin was treated with 1% TFA (v/v) in DCM for 2 min and then the solution filtrated off; this procedure was repeated 12 times. Then, glutamic acid was coupled using 4 eq of Fmoc-Glu(OtBu)-OH, 4 eq of (2-(1H-benzotriazol-1-yl)-1,1,3,3-tetramethyluronium hexafluorophosphate (HBTU), 4 eq of hydroxybenzotriazole (HOBt) and 8 eq of N,N-diisopropylethylamine (DIPEA). Deprotection of the Fmoc protecting group was achieved by treatment with 5 mL of 20% (v/v) piperidine in DMF, for 10 min for 2 times. For the pteroic acid coupling, 2 eq of pteroic acid were suspended in 10 mL of DMSO and heated at 50 °C for 1 h. 8 eq of DIPEA and 2 eq of benzotriazol-1-yl-

oxytripyrrolidinophosphonium hexafluorophosphate (PyBOP) were added to the suspension which was then reacted with the peptidyl-resin for 16 h, at 40 °C. The pteroic acid coupling was then repeated for a further 16 h, at 40 °C. Cleavage from the resin was performed in TFA/H2O/TIS 95:2.5:2.5 solution for 2 h. The resin was removed by filtration and the filtrate concentrated by N2 flow. The crude product was precipitated with cold diethyl ether, centrifuged (4,000 g for 10 min) and the supernatant discarded. The precipitate was dissolved in 2 mL of CH3CN + 0.1% TFA, diluted with 13 mL of H2O + 0.1% TFA, filtrated through a 0.22 µm syringe filter and purified by RP-HPLC. Lyophilization of the pure product fractions afforded the desired compound as a pale-yellow powder, which was characterized by LC-MS (Figure S18-S20). Yield 13-10%

2. Supplementary Figures

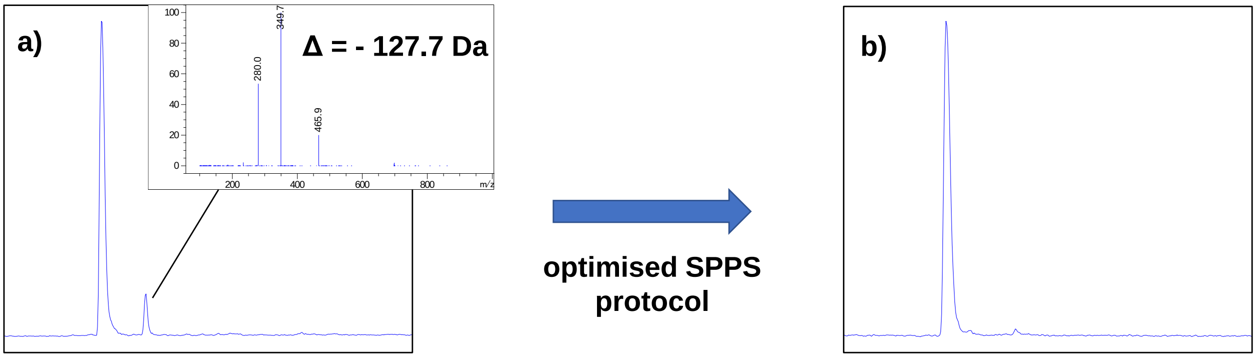

Figure S1. TIC chromatograms of the crude of compound **2** synthesis a) before and b) after optimizing the SPPS protocol.

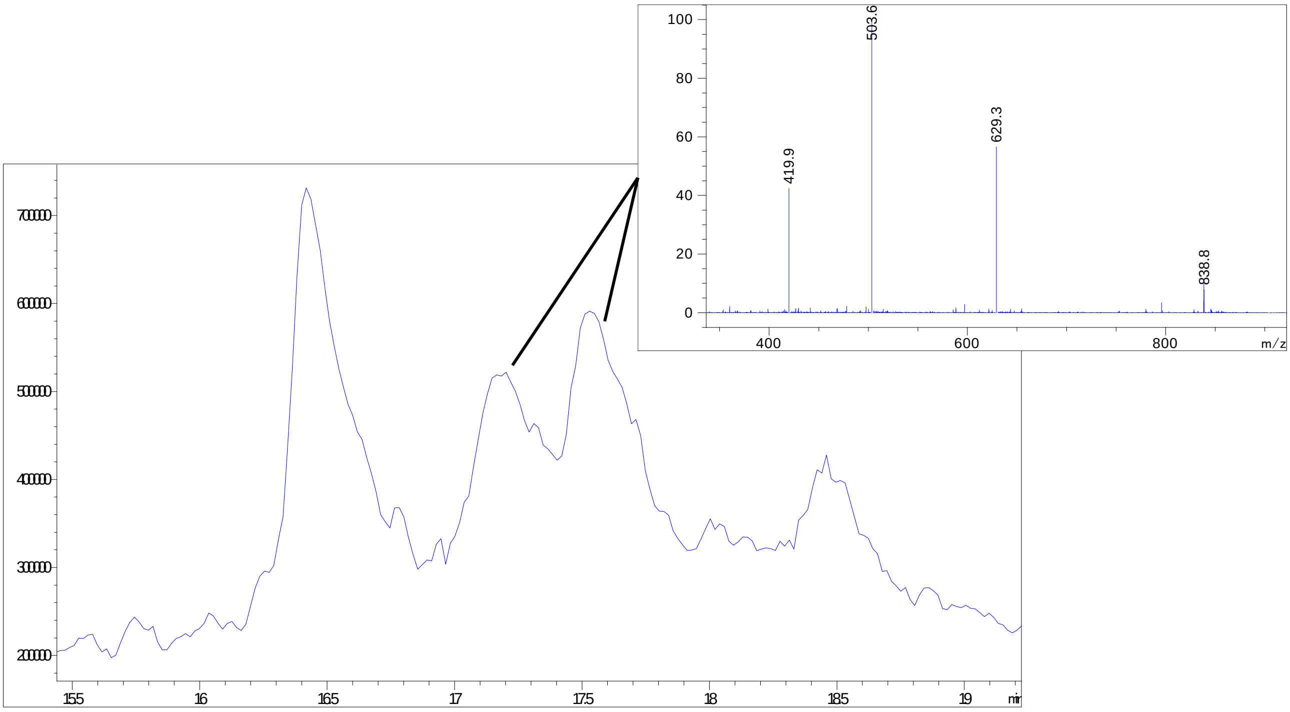

Figure S2. TIC chromatogram of the crude for the synthesis of compound **8** after coupling folate to the ε-amino group of the N-terminal lysine. Mass spectrum shows the same m/z peaks for two different chromatogram peaks, indicating the formation of two folate-labelled isomers.

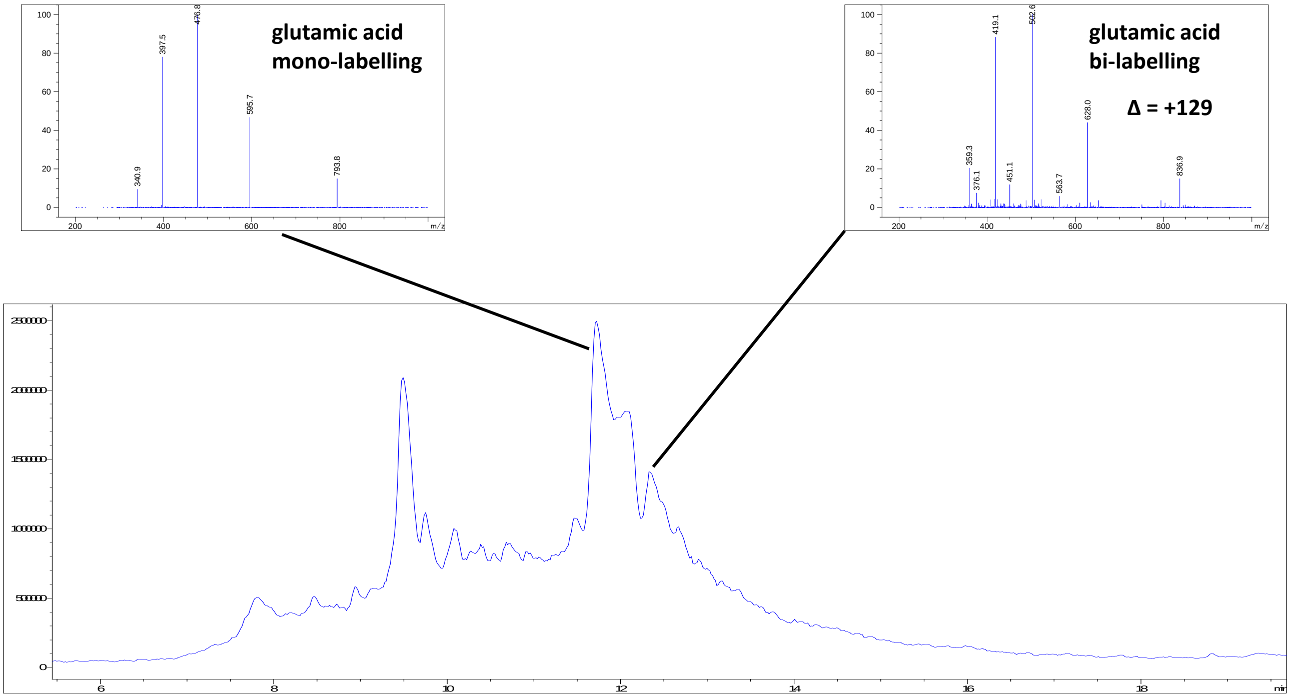

Figure S3. TIC chromatogram of the crude of synthesis for compound **10** after coupling and deprotection of glutamic acid to the ε-amino group of the N-terminal lysine. Mass spectrum shows the desired m/z for the mono-labelled glutamic acid intermediate (on the left) along with the bi-labelled glutamic acid intermediate (on the right). This led to an inseparable mixture of the desired product and bi-labelled folate side product.

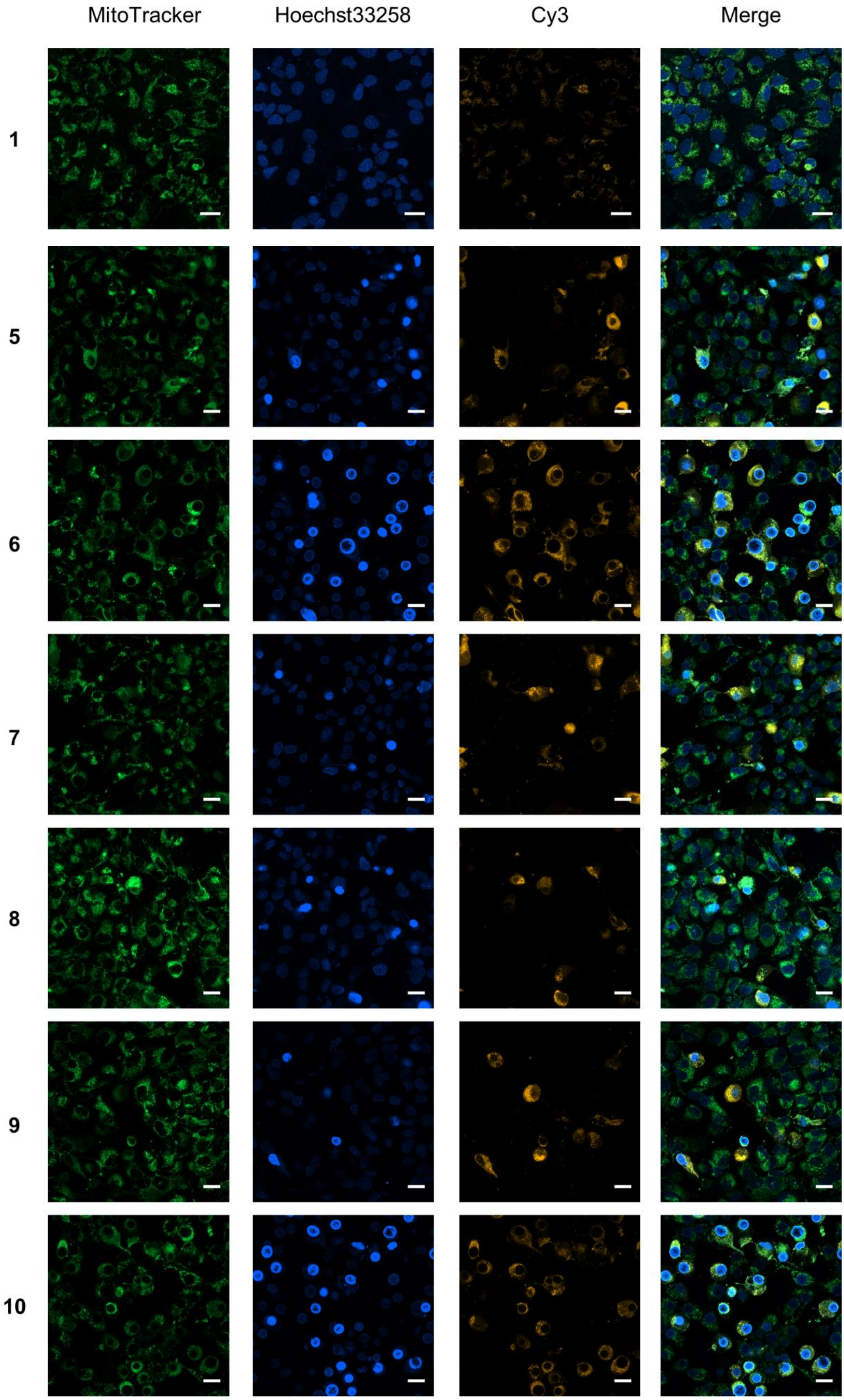

Figure S4. Confocal microscopy images of SK-OV-3 cells treated with Cy3-containing conjugates. Cells were treated with 10 μM of the indicated conjugate for 10 min at 37 °C, washed, stained with 50 nM MitoTracker Green and 10 μg/ml Hoechst33258 for 10 min at 37 °C, washed and imaged at 20X. Pearson's correlation coefficients of MitoTracker Green and Cy3 fluorescence for compounds **1**, **5**, **6**, **7**, **8**, **9** and **10** are 0.84, 0.76, 0.74, 0.74, 0.53, 0.52 and 0.79, respectively.

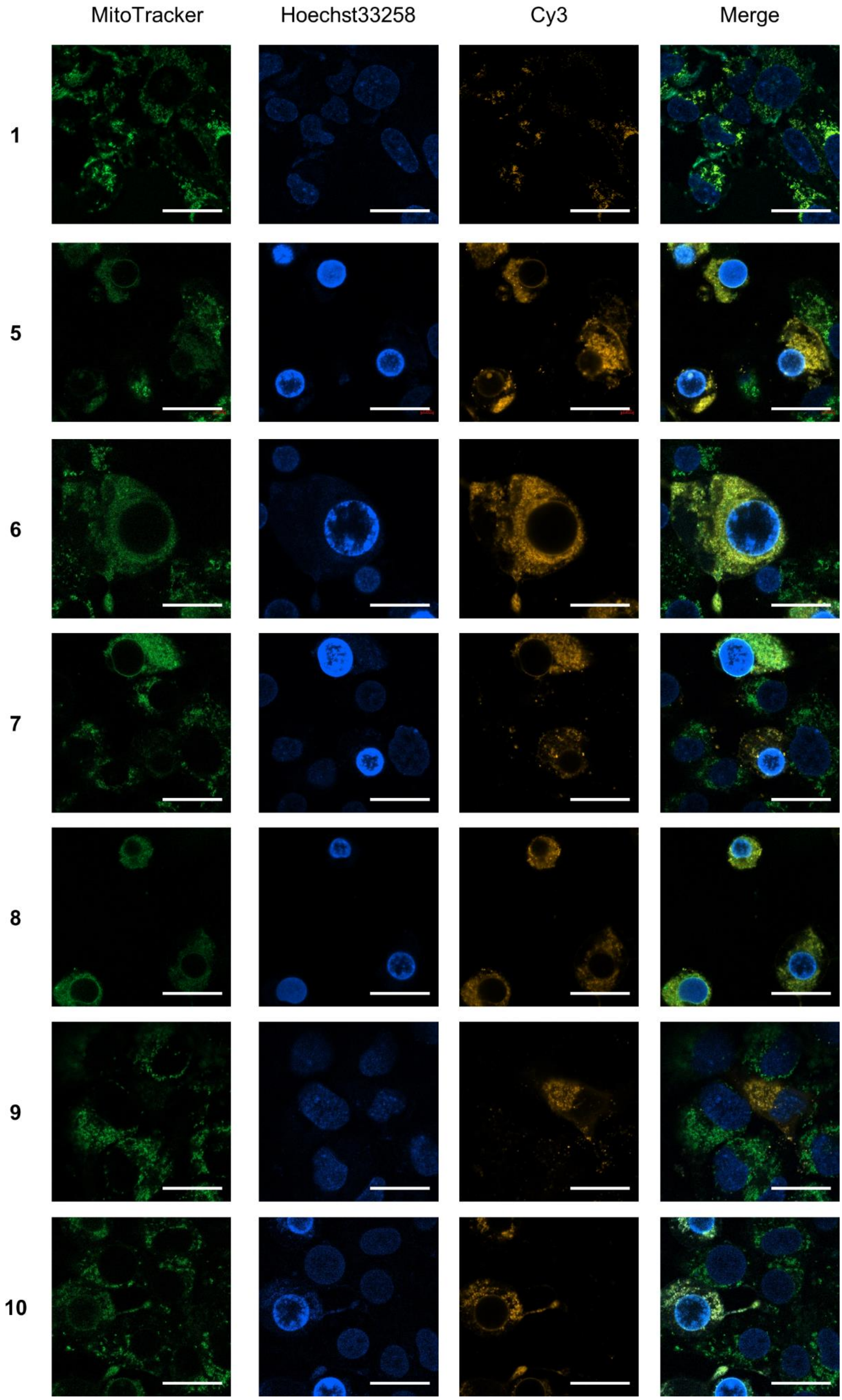

Figure S5. Confocal microscopy images of SK-OV-3 cells treated with Cy3-containing conjugates. Cells were treated with 10 μM of the indicated conjugate for 10 min at 37 °C, washed, stained with 50 nM MitoTracker Green and 10 μg/ml Hoechst33258 for 10 min at 37 °C, washed and imaged at 63X. Pearson's correlation coefficients of MitoTracker Green and Cy3 fluorescence for compounds **1**, **5**, **6**, **7**, **8**, **9** and **10** are 0.75, 0.73, 0.91, 0.78, 0.85, 0.43 and 0.78, respectively.

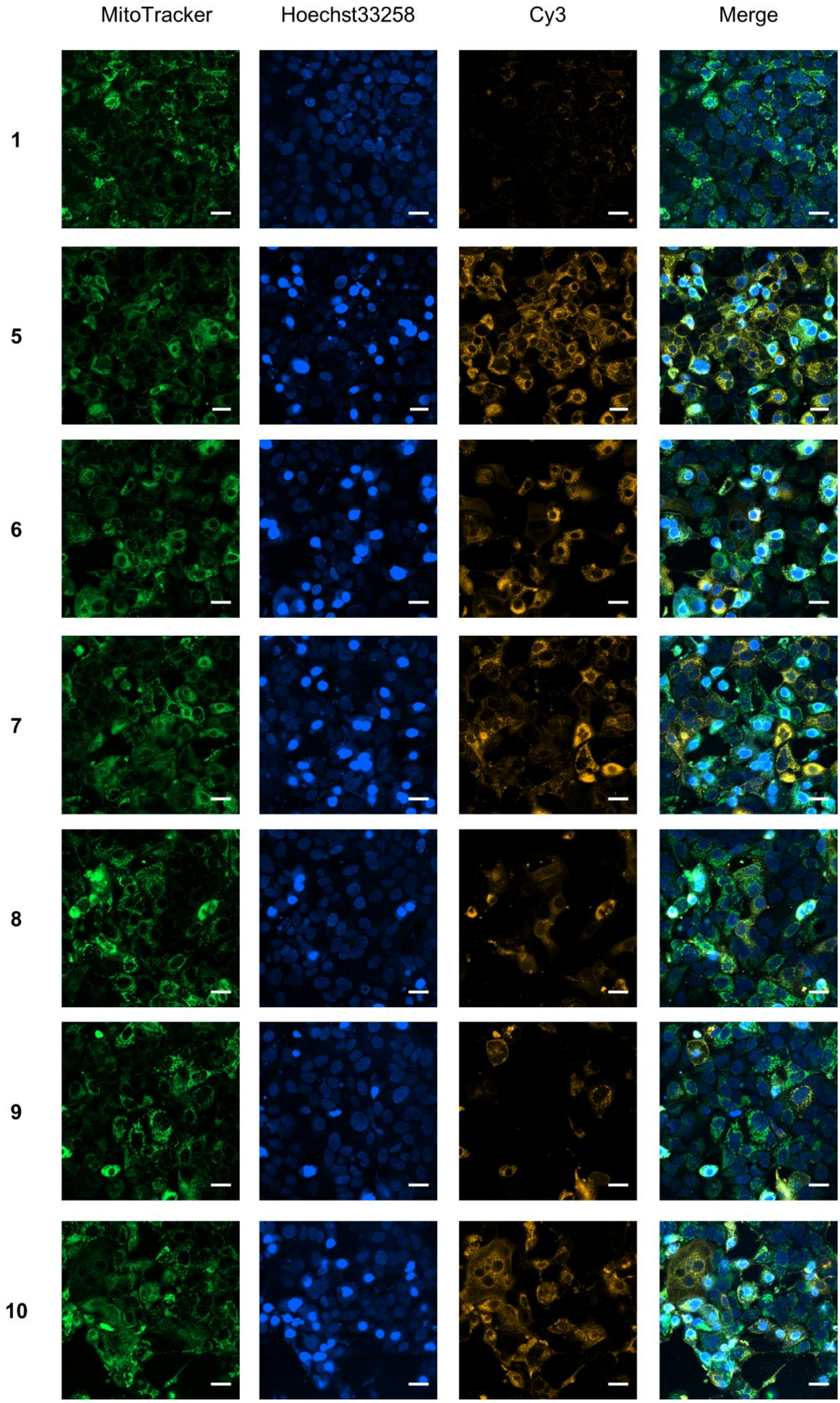

Figure S6. Confocal microscopy images of MCF7 cells treated with Cy3-containing conjugates. Cells were treated with 10 μM of the indicated conjugate for 10 min at 37 °C, washed, stained with 50 nM MitoTracker Green and 10 μg/ml Hoechst33258 for 10 min at 37 °C, washed and imaged at 20X. Pearson's correlation coefficients of MitoTracker Green and Cy3 fluorescence for compounds **1**, **5**, **6**, **7**, **8**, **9** and **10** are 0.52, 0.80, 0.81, 0.52, 0.60, 0.61 and 0.68, respectively.

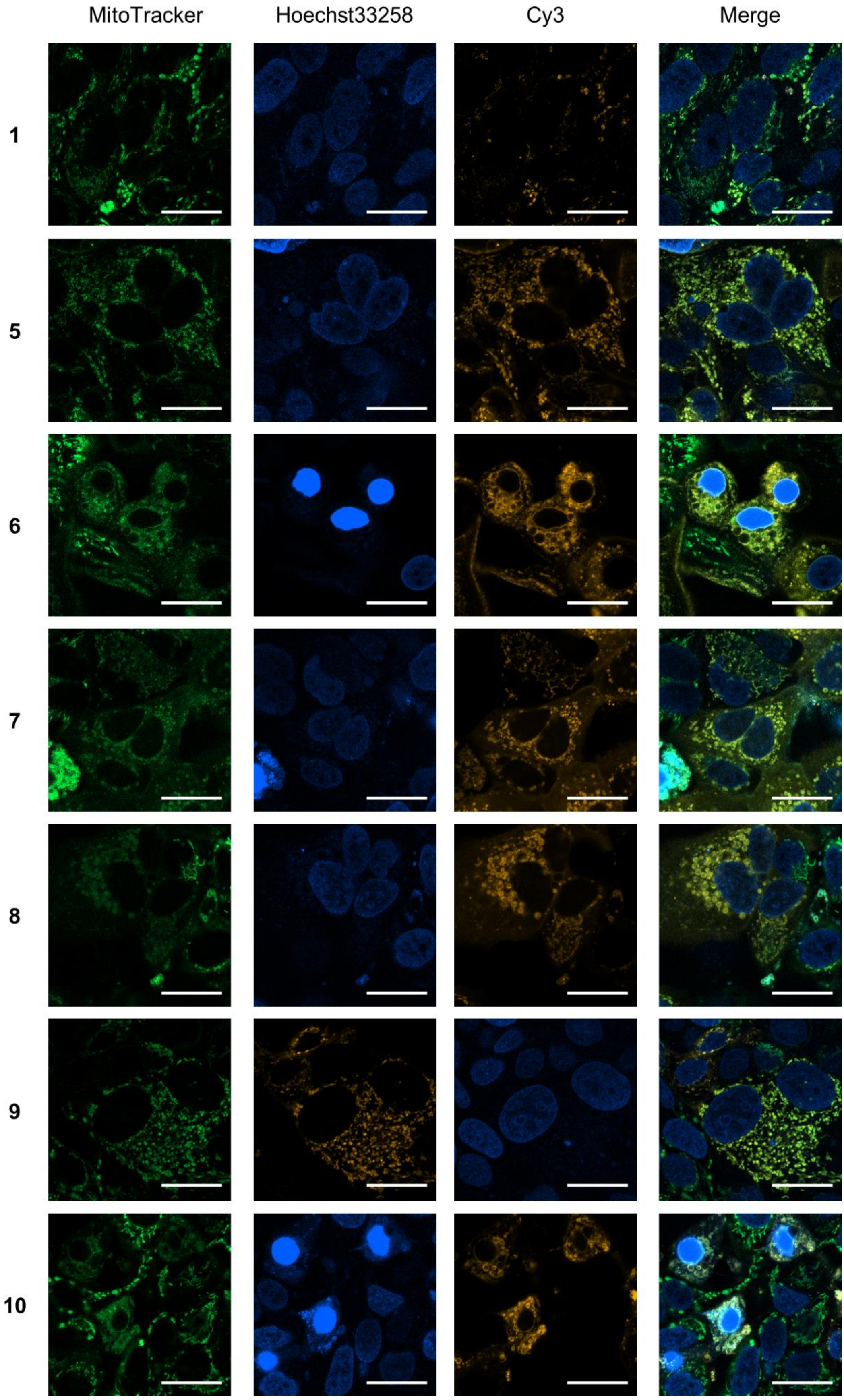

Figure S7. Confocal microscopy images of MCF7 cells treated with Cy3-containing conjugates. Cells were treated with 10 μM of the indicated conjugate for 10 min at 37 °C, washed, stained with 50 nM MitoTracker Green and 10 μg/ml Hoechst33258 for 10 min at 37 °C, washed and imaged at 63X. Pearson's correlation coefficients of MitoTracker Green and Cy3 fluorescence for compounds **1**, **5**, **6**, **7**, **8**, **9** and **10** are 0.64, 0.81, 0.61, 0.63, 0.69, 0.82 and 0.60, respectively.

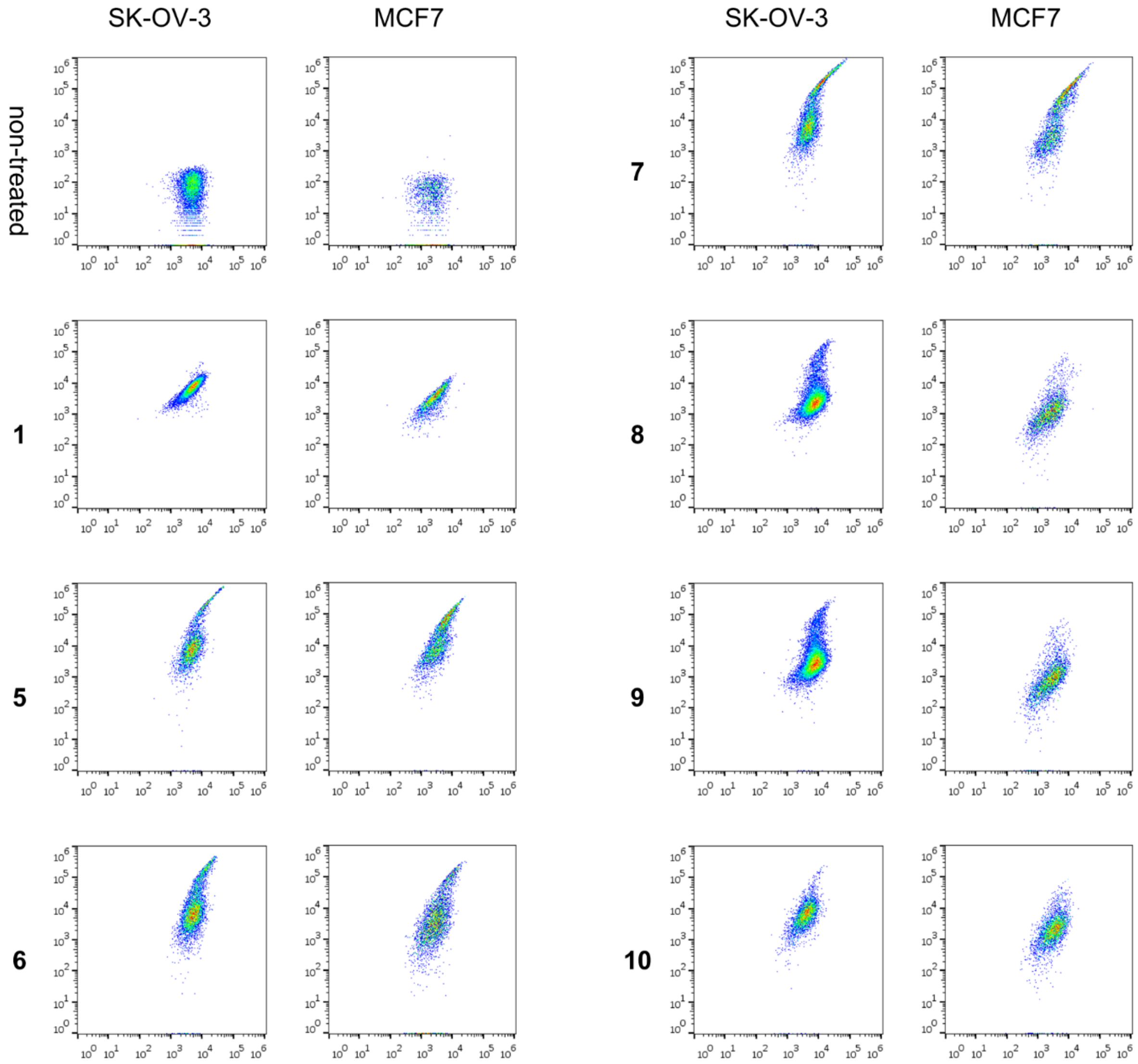

Figure S8. Representative flow cytometry scatter plots of Cy3-containing conjugate uptakes by SK-OV-3 and MCF7 cells. Cells were treated with 10 µM of the indicated conjugate for 10 min at 37 °C, washed, stained with 50 nM MitoTracker Green for 10 min at 37 °C, washed, trypsinized and subjected to flow cytometry analysis. X- and y-axes show signals of MitoTracker and Cy3, respectively.

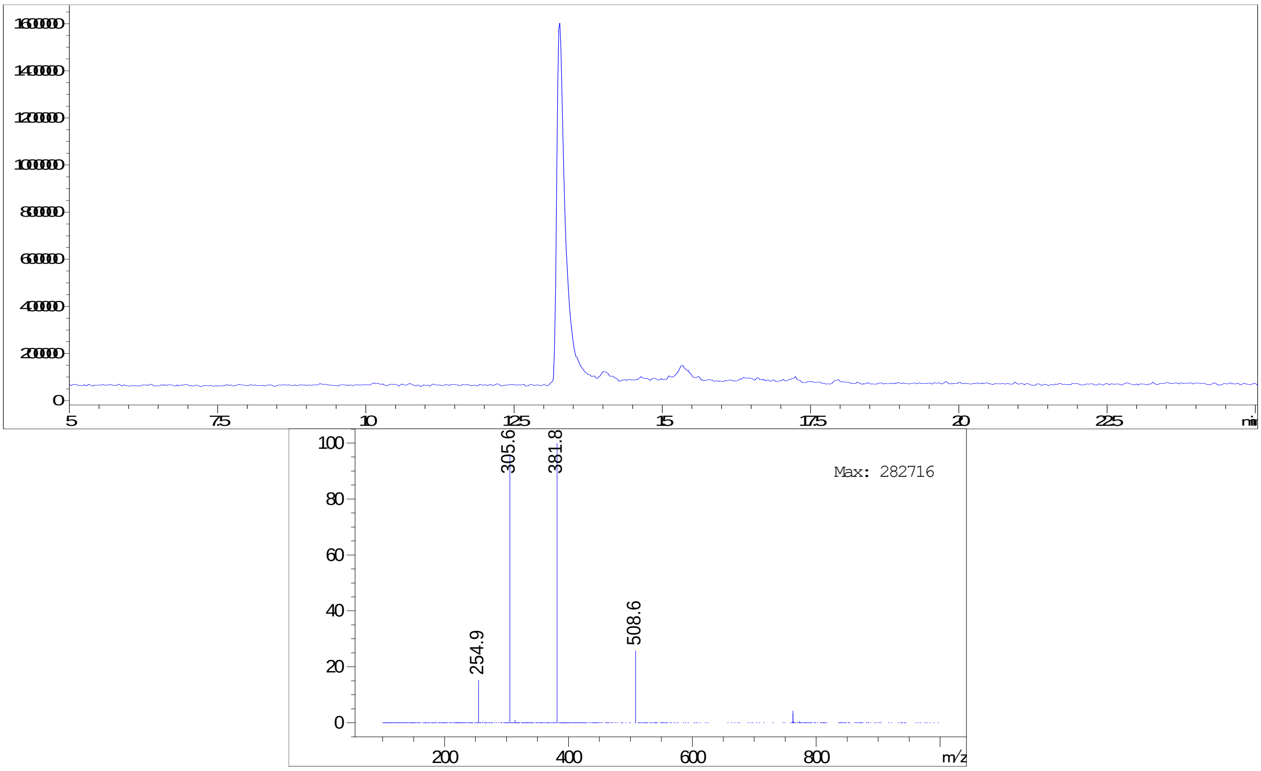

Figure S9. TIC chromatogram (+ mass spectrum at 13.181:13.509 min) of compound **2.** Column: *Poroshell 120 EC-C18*, 2.1 x 100 mm, 4 µm particle size, 120 Å pore size; Method: flow rate = 0.5 mL · min^{-1}, H_2O: CH_3CN, 0.1% HCOOH, 95:5 for 2 min → 95:5 to 70:30 over 25 min → 5:95 for 5 min.

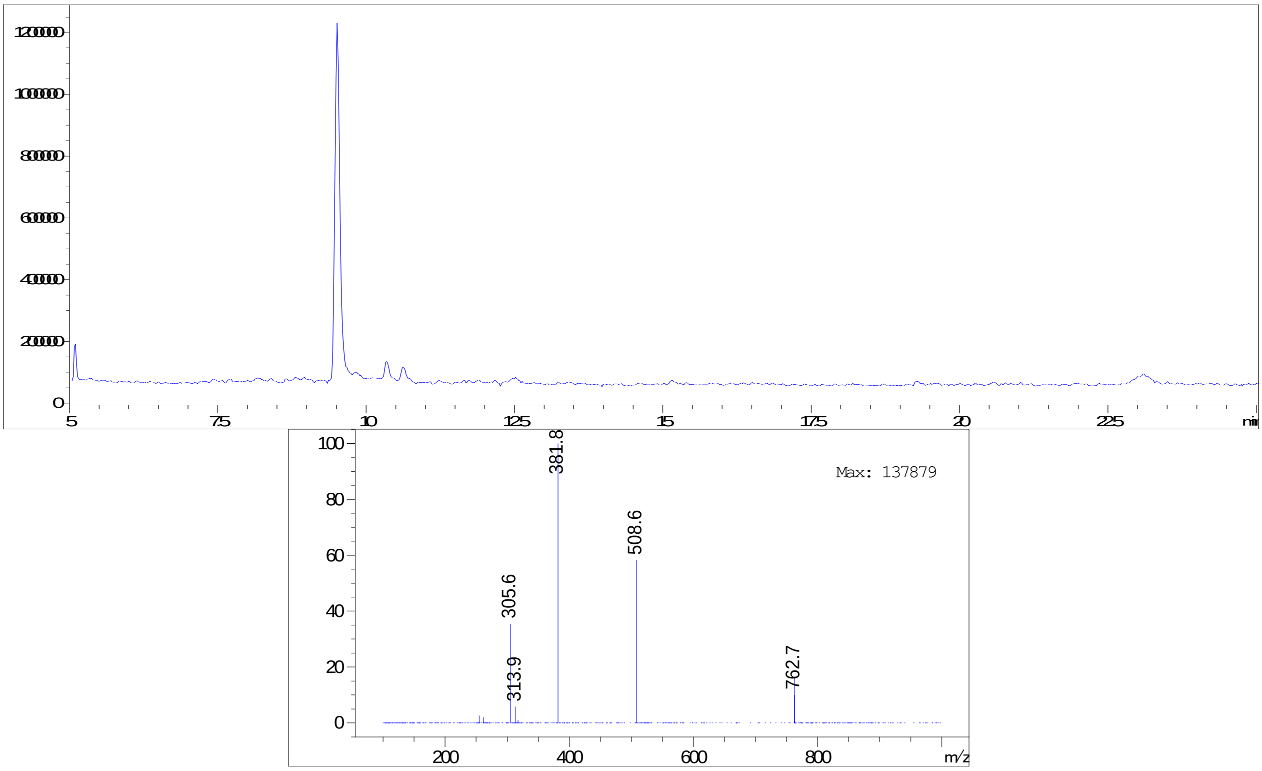

Figure S10. TIC chromatogram (+ mass spectrum at 9.439:9.639 min) of compound **3.** Column: *Poroshell 120 EC-C18*, 2.1 x 100 mm, 4 µm particle size, 120 Å pore size; Method: flow rate = 0.5 mL · min^{-1}, H_2O: CH_3CN, 0.1% HCOOH, 95:5 for 2 min → 95:5 to 40:60 over 23 min → 5:95 for 5 min.

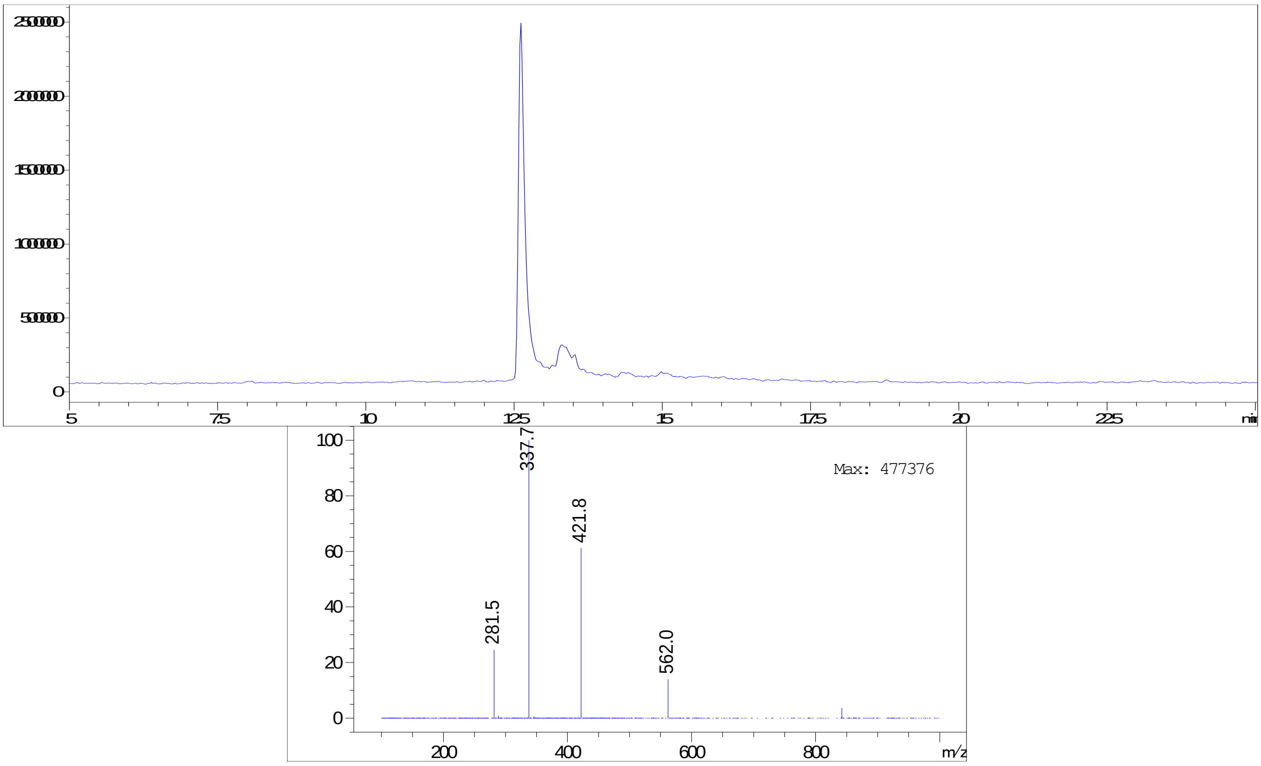

Figure S11. TIC chromatogram (+ mass spectrum at 12.562:12.853 min) of compound **4.** Column: *Poroshell 120 EC-C18*, 2.1 x 100 mm, 4 µm particle size, 120 Å pore size; Method: flow rate = 0.5 mL · min^{-1}, H_2O: CH_3CN, 0.1% HCOOH, 95:5 for 2 min → 95:5 to 40:60 over 23 min → 5:95 for 5 min.

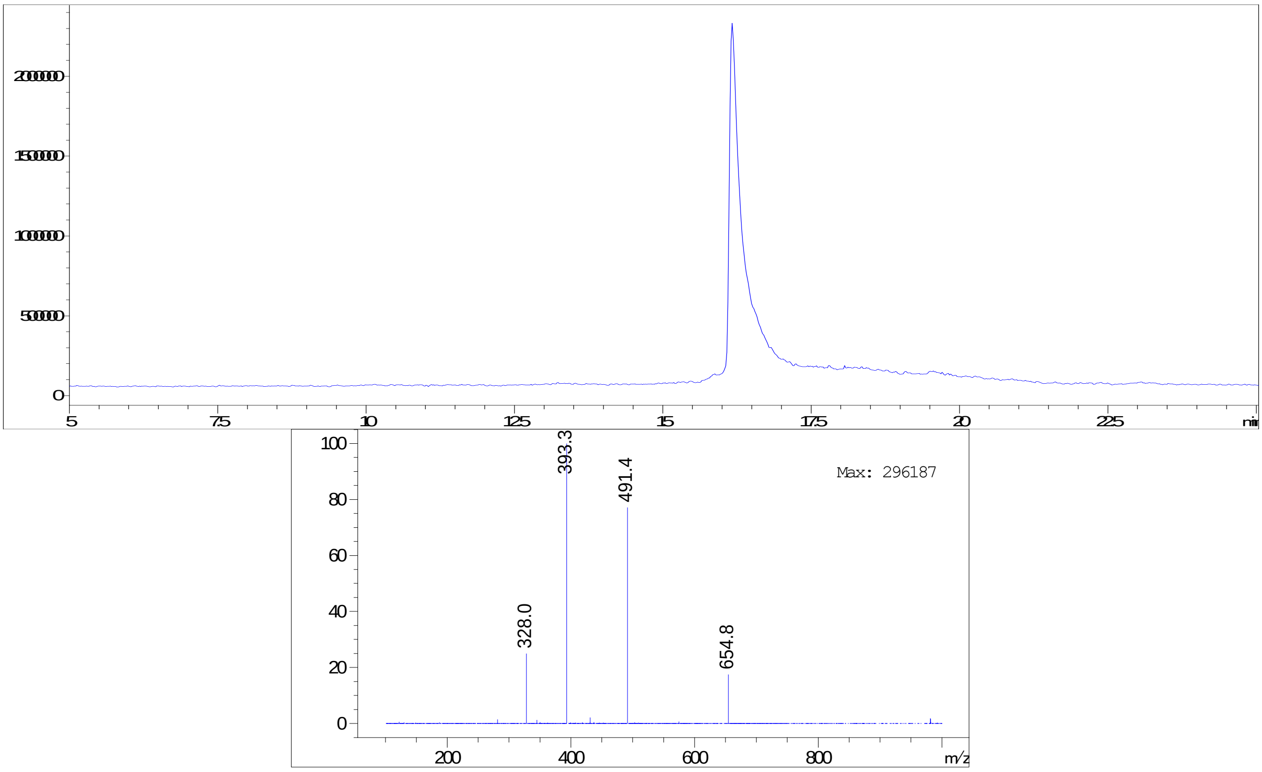

Figure S12. TIC chromatogram (+ mass spectrum at 16.042:16.771 min) of compound **5.** Column: *Poroshell 120 EC-C18*, 2.1 x 100 mm, 4 µm particle size, 120 Å pore size; Method: flow rate = 0.5 mL · min^{-1}, H_2O: CH_3CN, 0.1% HCOOH, 95:5 for 2 min → 95:5 to 40:60 over 23 min → 5:95 for 5 min.

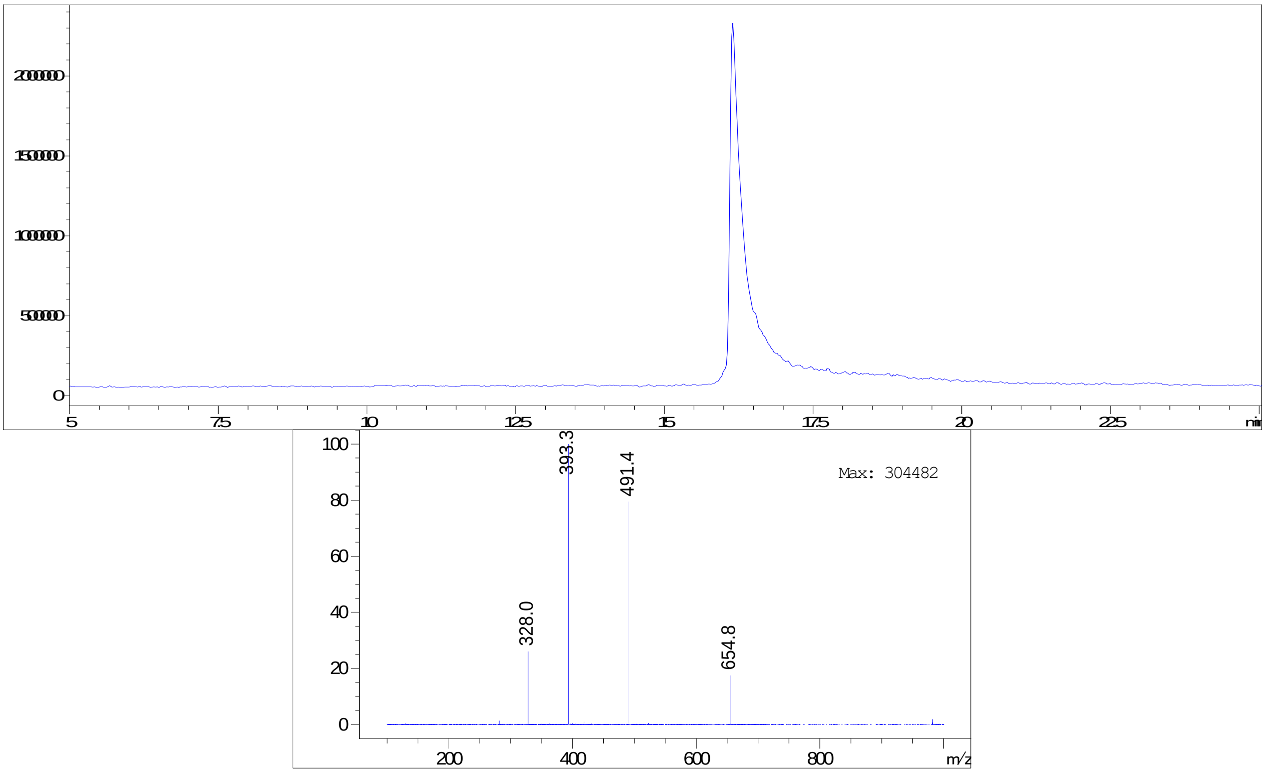

Figure S13. TIC chromatogram (+ mass spectrum at 16.188:16.680 min) of compound **6.** Column: *Poroshell 120 EC-C18*, 2.1 x 100 mm, 4 µm particle size, 120 Å pore size; Method: flow rate = 0.5 mL · min^{-1}, H_2O: CH_3CN, 0.1% HCOOH, 95:5 for 2 min → 95:5 to 40:60 over 23 min → 5:95 for 5 min.

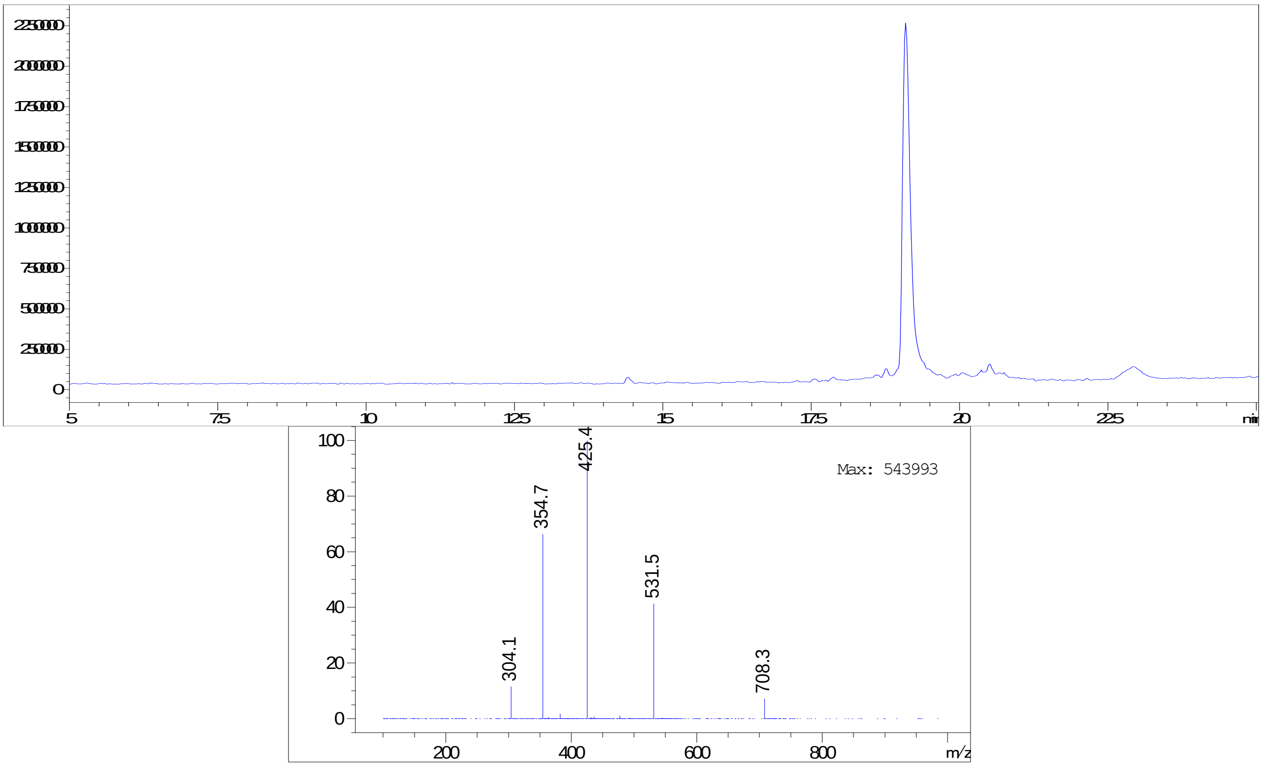

Figure S14. TIC chromatogram (+ mass spectrum at 19.056:19.256 min) of compound **7.** Column: *ACE 3 C18*, 2.1 x 100 mm, 3 µm particle size, 100 Å pore size; Method: flow rate = 0.3 mL · min^{-1}, H_2O: CH_3CN, 0.1% HCOOH, 85:15 to 5:95 over 35 min → 5:95 for 2 min.

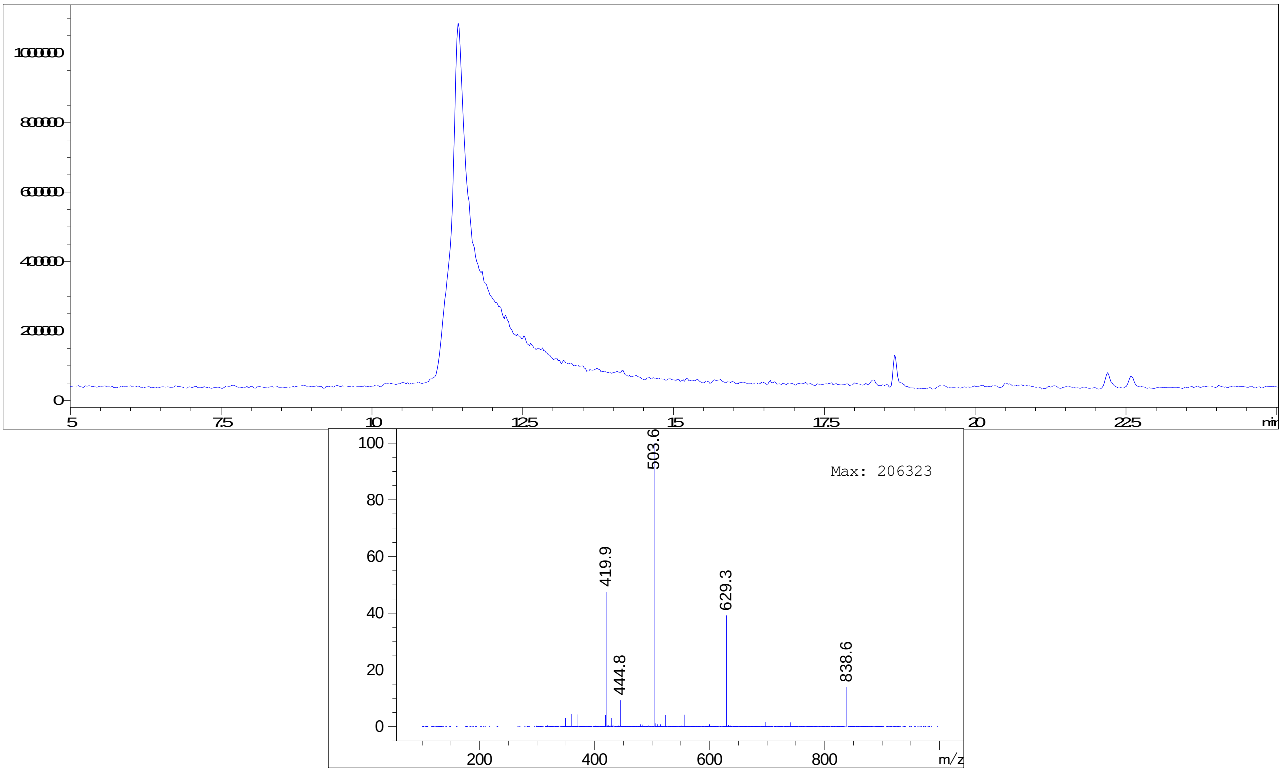

Figure S15. TIC chromatogram (+ mass spectrum at 11.300:11.719 min) of compound **8.** Column: *ACE 3 C18*, 2.1 x 100 mm, 3 µm particle size, 100 Å pore size; Method: flow rate = 0.3 mL · min^{-1}, H_2O: CH_3CN, 0.1% HCOOH, 85:15 for 2 min → 85:15 to 40:60 over 13 min → 5:95 for 7 min → 5:95 to 85:15 over 0.5 min → 85:15 for 7 min.

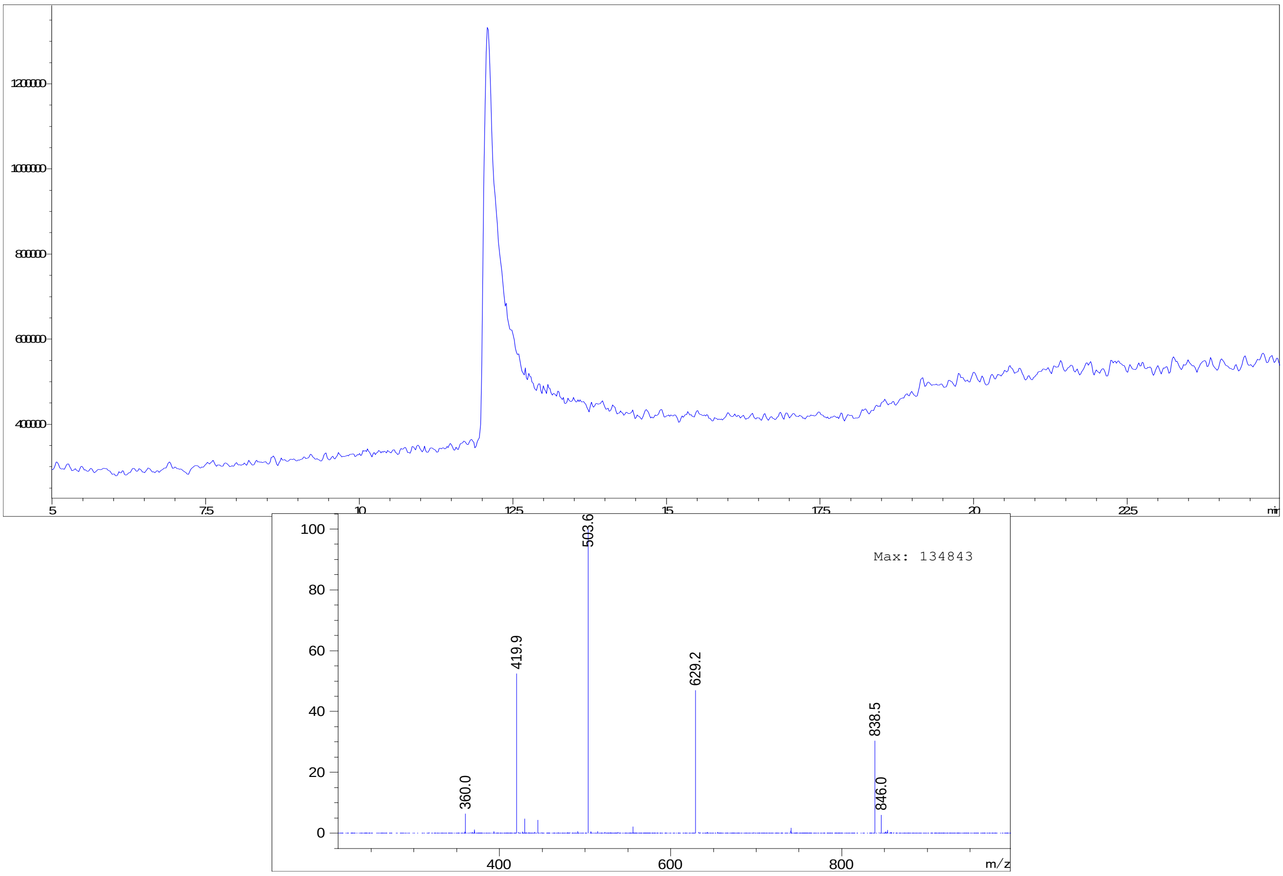

Figure S16. TIC chromatogram (+ mass spectrum at 11.956:12.667 min) of compound **9.** Column: *ACE 3 C18*, 2.1 x 100 mm, 3 µm particle size, 100 Å pore size; Method: flow rate = 0.3 mL · min^{-1}, H_2O: CH_3CN, 0.1% HCOOH, 85:15 for 2 min → 85:15 to 40:60 over 13 min → 5:95 for 7 min → 5:95 to 85:15 over 0.5 min → 85:15 for 7 min.

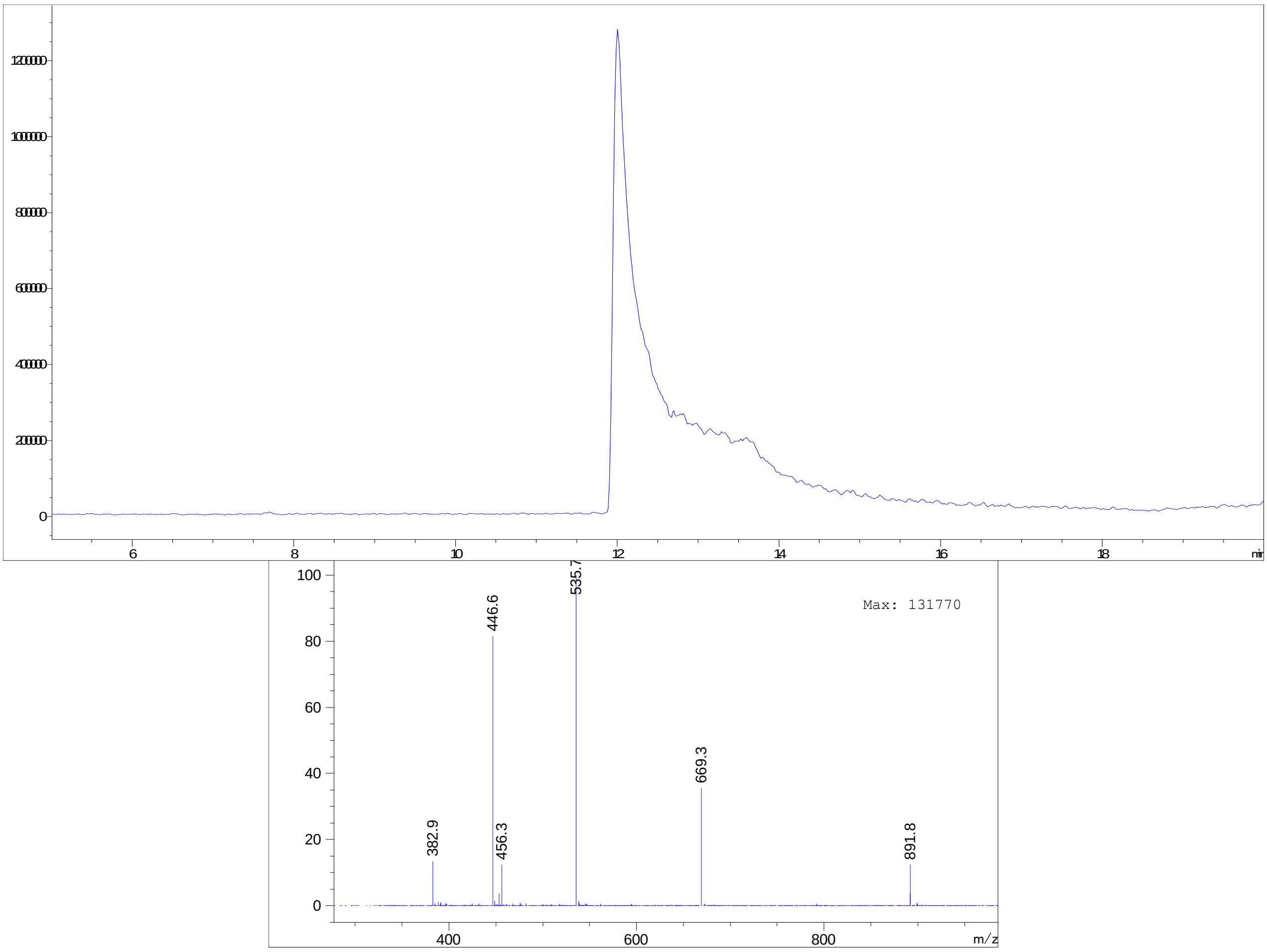

Figure S17. TIC chromatogram (+ mass spectrum at 11.985:12.654 min) of compound **10.** Column: *ACE 3 C18*, 2.1 x 100 mm, 3 µm particle size, 100 Å pore size; Method: flow rate = 0.3 mL · min^{-1}, H_2O: CH_3CN, 0.1% HCOOH, 85:15 for 2 min → 85:15 to 40:60 over 13 min → 5:95 for 7 min → 5:95 to 85:15 over 0.5 min → 85:15 for 7 min.

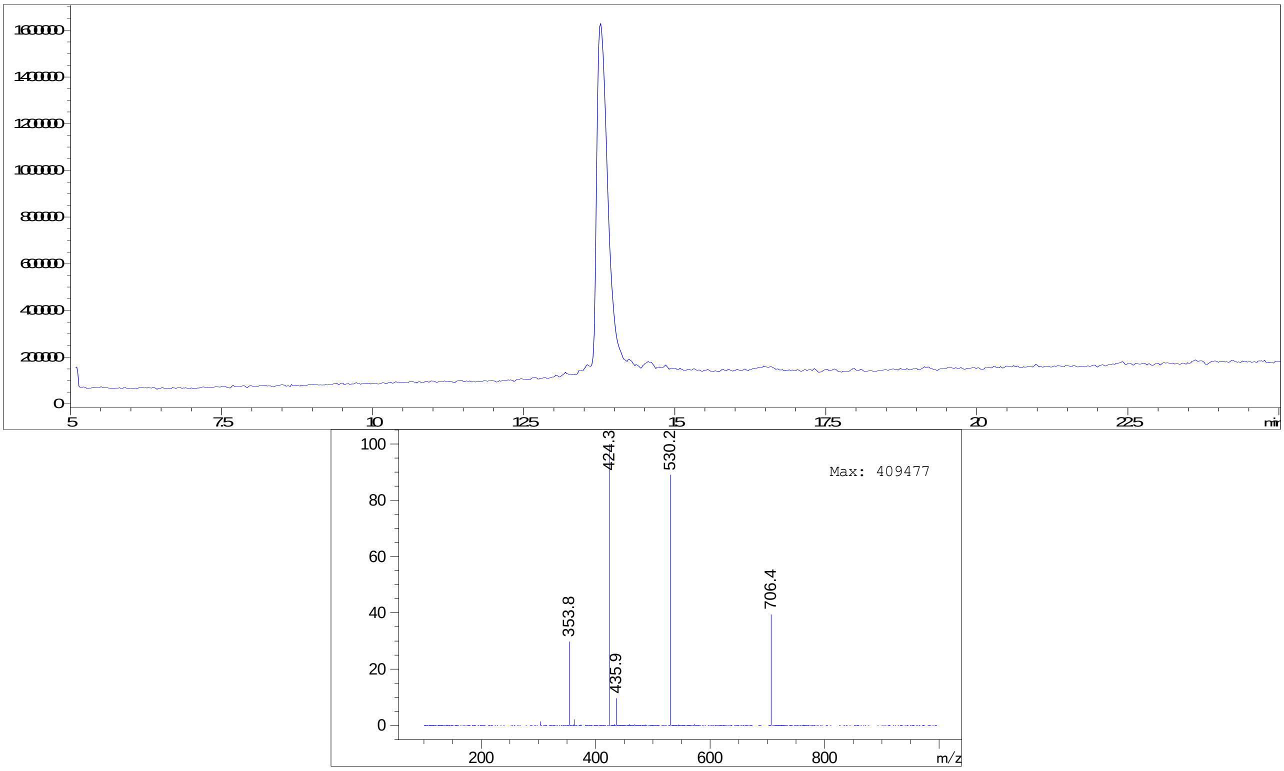

Figure S18. TIC chromatogram (+ mass spectrum at 13.721:13.940 min) of compound **11.** Column: *Poroshell 120 EC-C18*, 2.1 x 100 mm, 4 µm particle size, 120 Å pore size; Method: flow rate = 0.5 mL · min^{-1}, H_2O: CH_3CN, 0.1% HCOOH, 95:5 for 2 min → 95:5 to 40:60 over 23 min → 5:95 for 5 min.

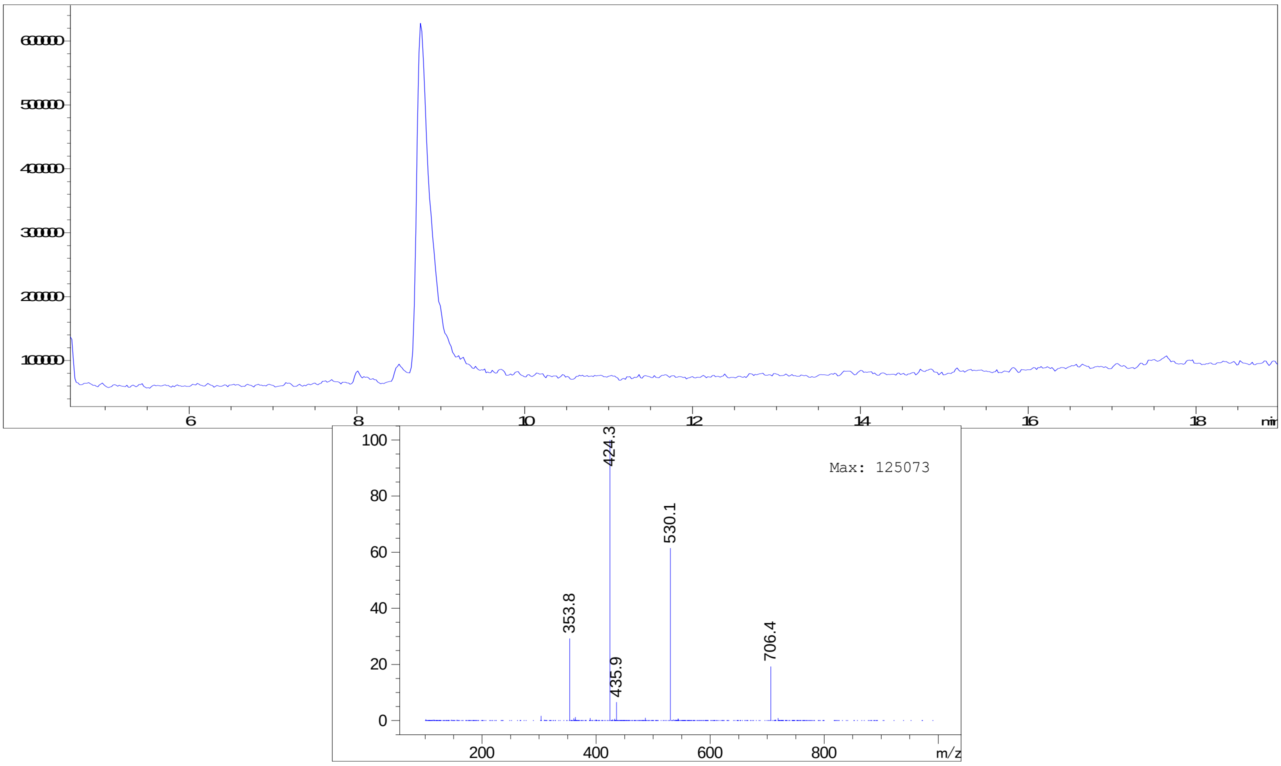

Figure S19. TIC chromatogram (+ mass spectrum at 8.684:9.012 min) of compound **12.** Column: *Poroshell 120 EC-C18*, 2.1 x 100 mm, 4 µm particle size, 120 Å pore size; Method: flow rate = 0.5 mL · min^{-1}, H_2O: CH_3CN, 0.1% HCOOH, 95:5 for 1 min → 95:5 to 45:65 over 15 min → 5:95 for 5 min.

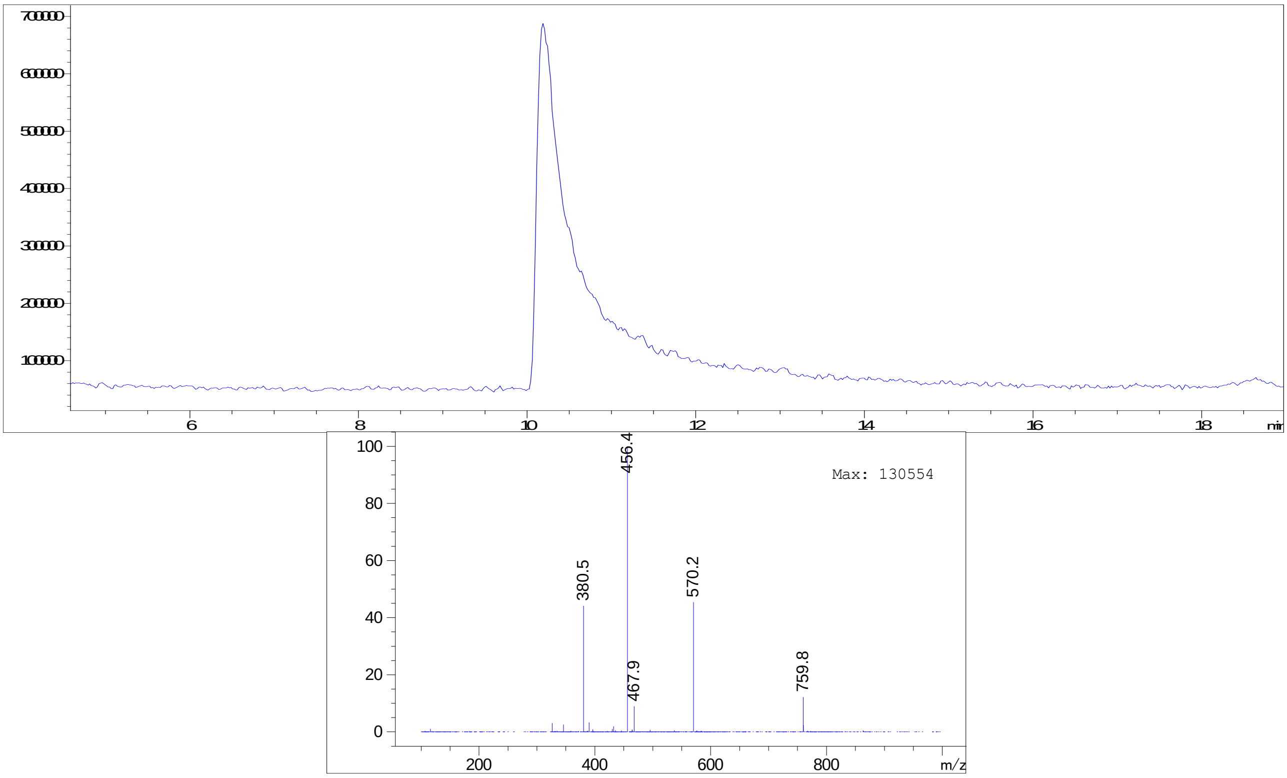

Figure S20. TIC chromatogram (+ mass spectrum at 10.097:10.735 min) of compound **13.** Column: *ACE 3 C18*, 2.1 x 100 mm, 3 µm particle size, 100 Å pore size; Method: flow rate = 0.3 mL · min^{-1}, H_2O: CH_3CN, 0.1% HCOOH, 85:15 for 2 min → 85:15 to 40:60 over 13 min → 5:95 for 7 min → 5:95 to 85:15 over 0.5 min → 85:15 for 7 min.

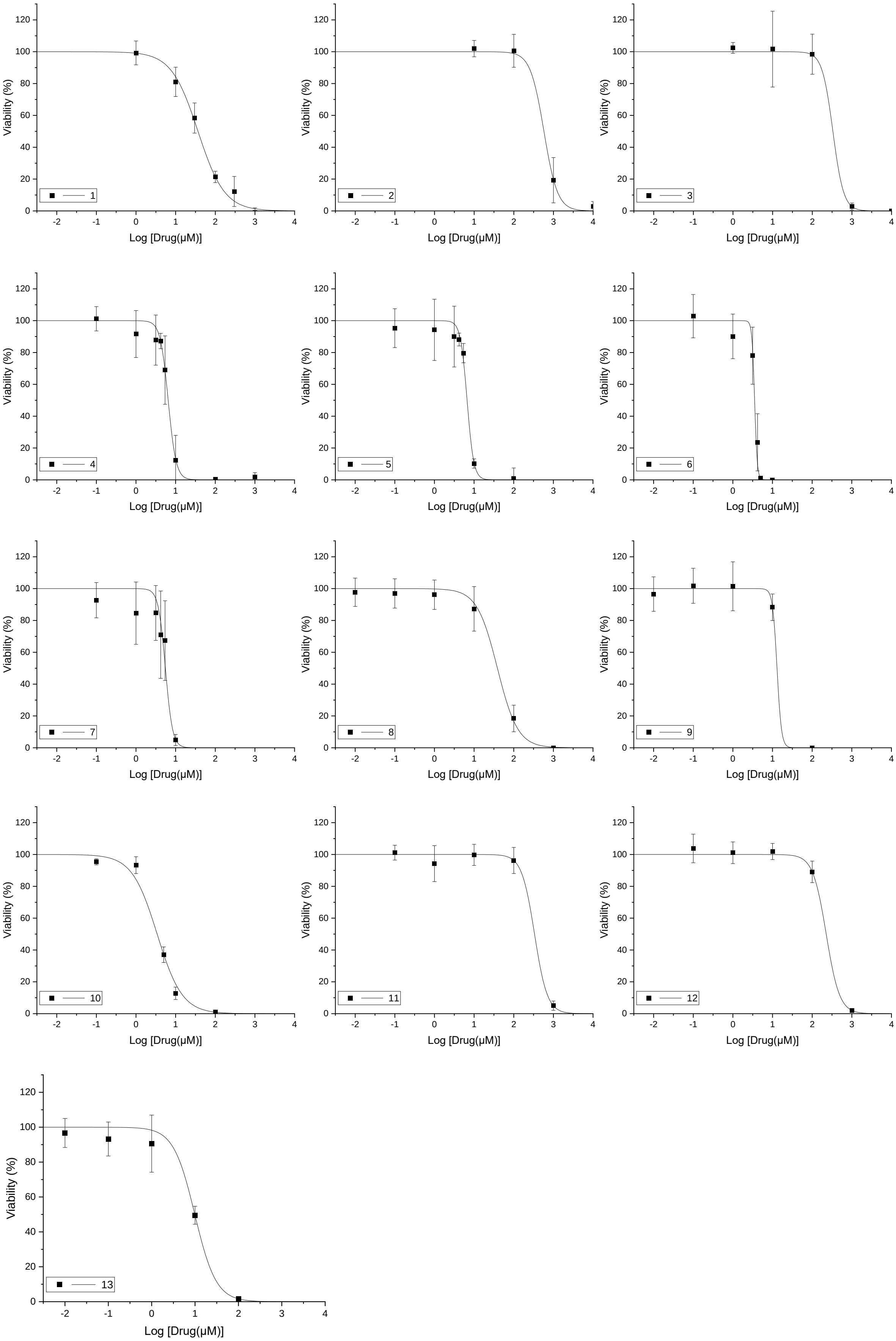

Figure S21. Cytotoxicity of compounds **1**–**13** towards KB cell lines. Fitting curves are shown as solid black lines. Dots and error bars represent the mean and the standard deviation from a minimum of nine values resulted from three biological replicates (i.e., cells split from three different passages); each biological replicate is calculated from three technical replicates (i.e., cells split from the same passage).

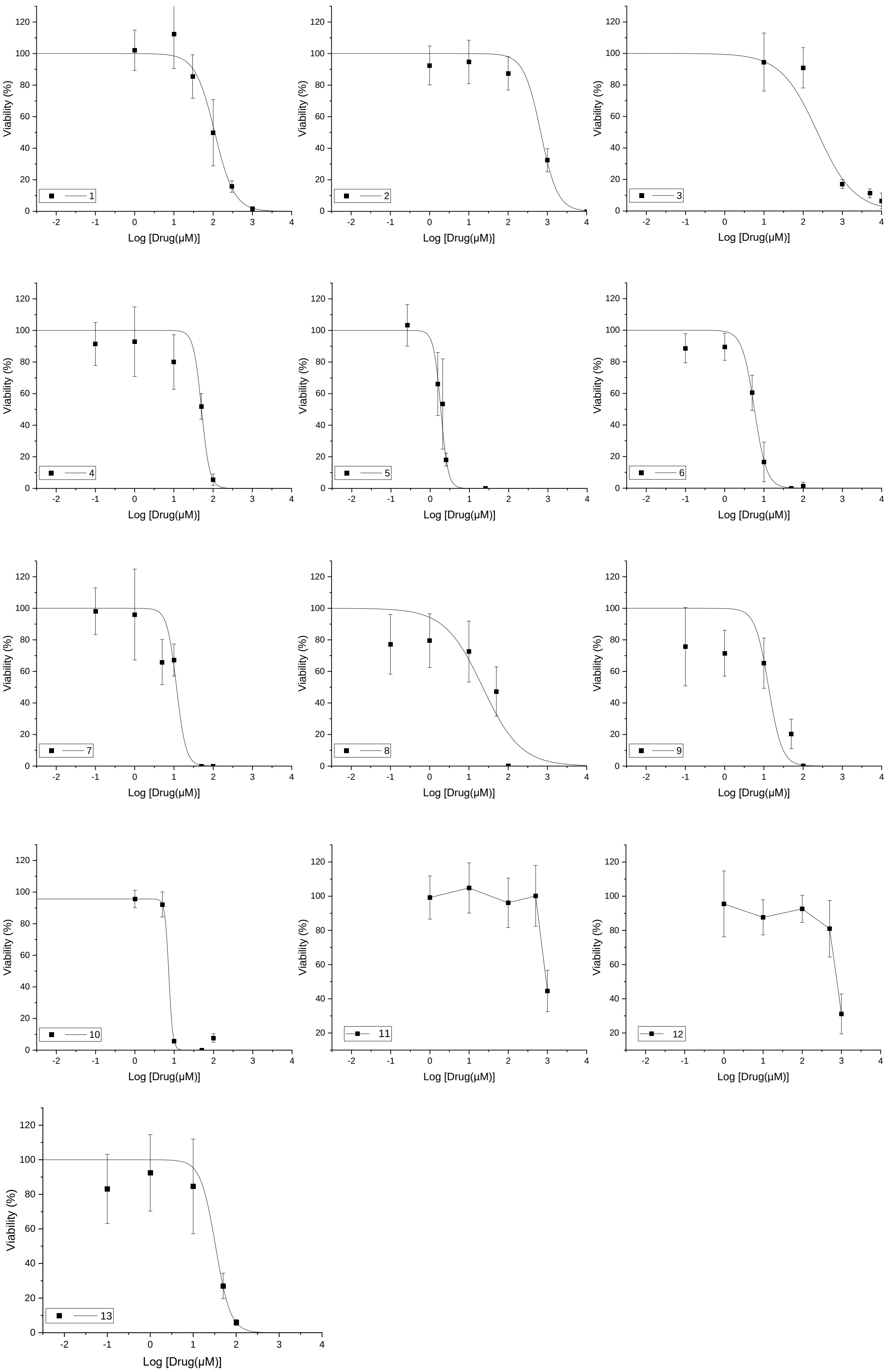

Figure S22. Cytotoxicity of compounds **1–13** towards MCF7 cell lines. Fitting curves are shown as solid black lines. Data for compounds **11** and **12** were not fitted. Dots and error bars represent the mean and the standard deviation from a minimum of nine values resulted from three biological replicates (i.e., cells split from three different passages); each biological replicate is calculated from three technical replicates (i.e., cells split from the same passage).

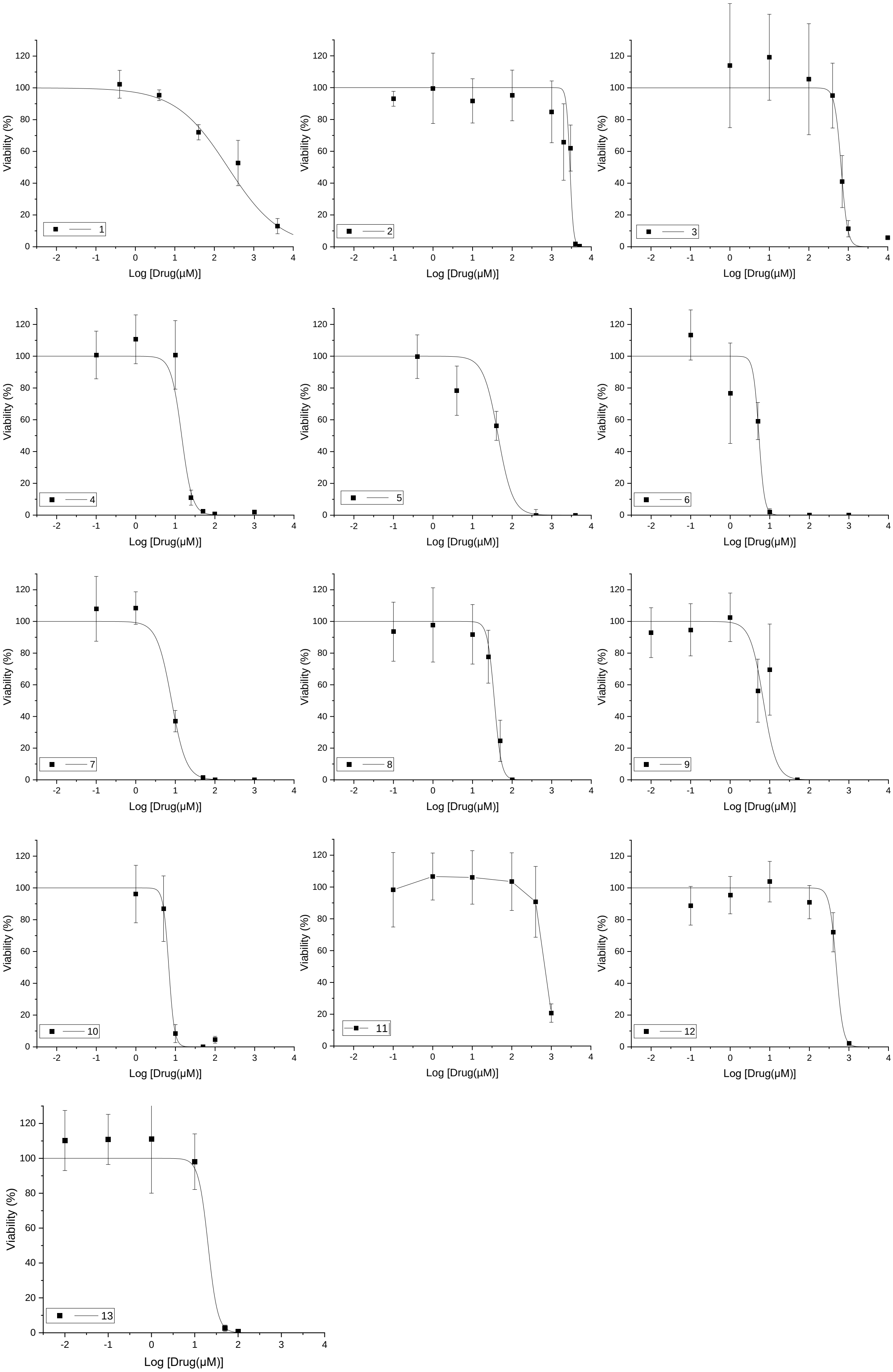

Figure S23. Cytotoxicity of compounds **1**–**13** towards HEK293 cell lines. Fitting curves are shown as solid black lines. Data for compound **11** were not fitted. Dots and error bars represent the mean and the standard deviation from a minimum of nine values resulted from three biological replicates (i.e., cells split from three different passages); each biological replicate is calculated from three technical replicates (i.e., cells split from the same passage).

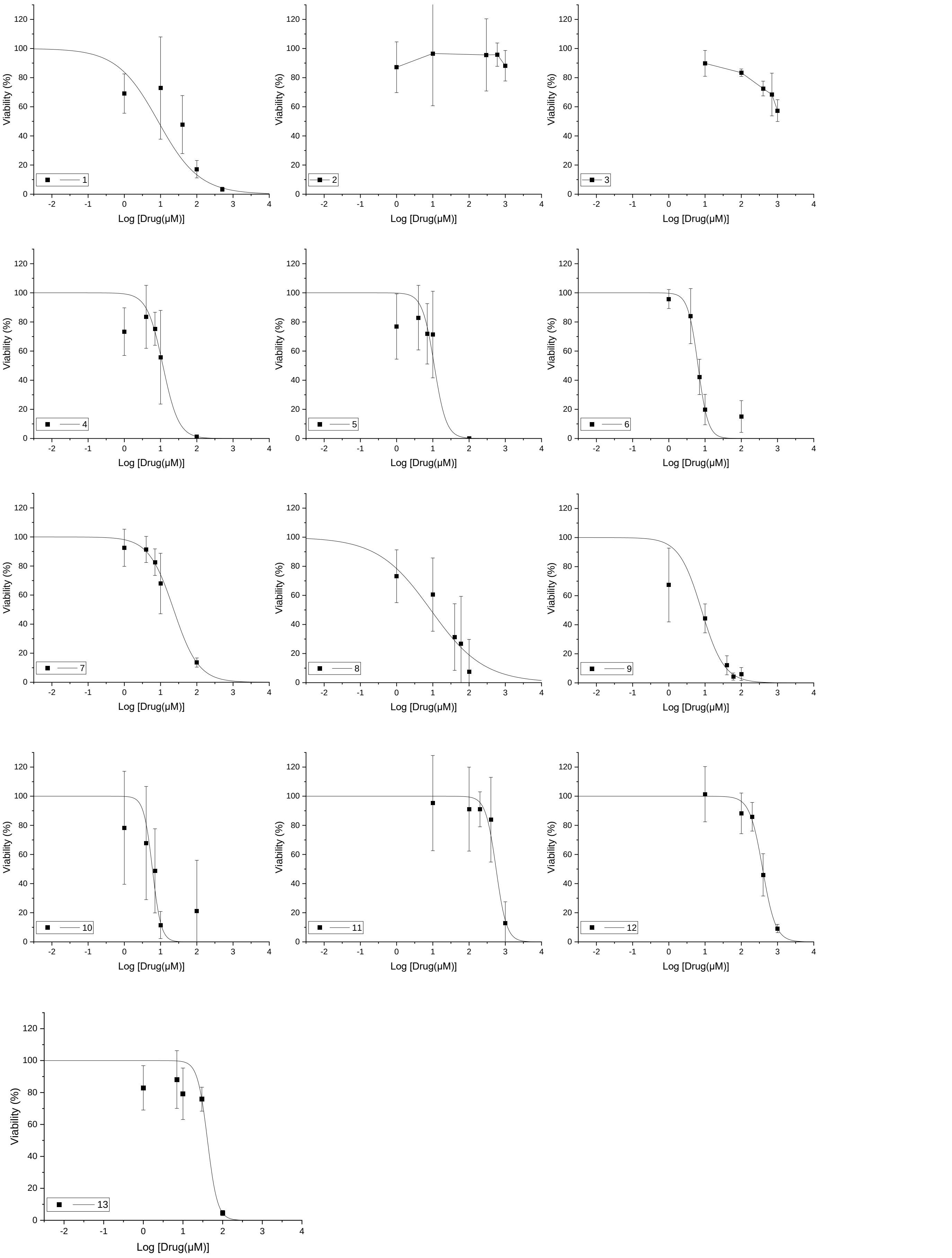

Figure S24. Cytotoxicity of compounds **1**–**13** towards SK-OV-3 cell lines. Fitting curves are shown as solid black lines. Data for compounds **2** and **3** were not fitted. Dots and error bars represent the mean and the standard deviation from a minimum of nine values resulted from three biological replicates (i.e., cells split from three different passages); each biological replicate is calculated from three technical replicates (i.e., cells split from the same passage).

3. ¹H-NMR and high-resolution mass spectrum of **1**

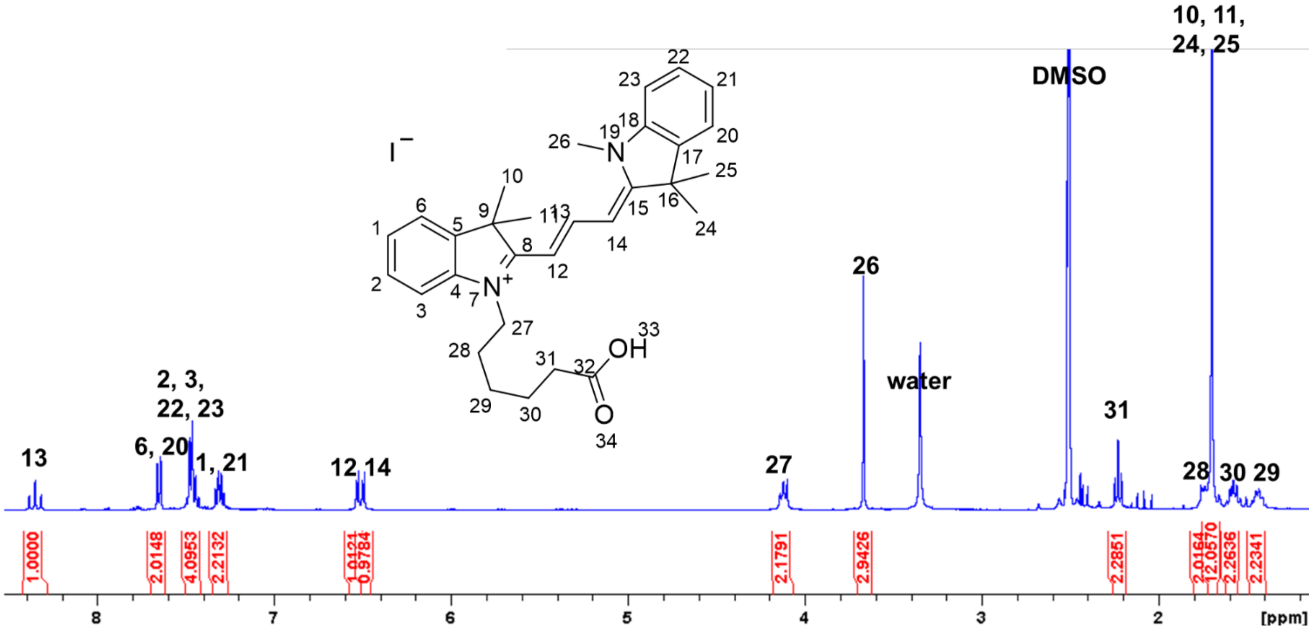

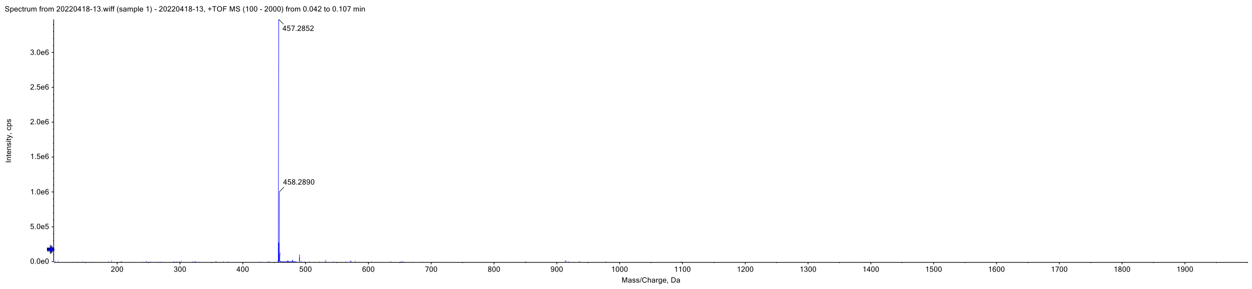

4. Table S1. Calculated and found mass to charge ratio for compounds **1–13** by mass spectroscopy.

Compound	Calculated m/z (z)	Found m/z (z)	Purity* (%)
**1**	457.2850 (+1)	457.2852 (+1)	95
**2**	762.0478 (+2)	762.0485 (+2)	94
**3**	762.0478 (+2)	762.0489 (+2)	93
**4**	561.7427 (+3)	561.7453 (+3)	99
**5**	654.7911 (+3)	654.7944 (+3)	99
**6**	654.7911 (+3)	654.7936 (+3)	100
**7**	708.1662 (+3)	708.1684 (+3)	98
**8**	838.5325 (+3)	838.5338 (+3)	89
**9**	838.5325 (+3)	838.5348 (+3)	98
**10**	891.9076 (+3)	891.9061 (+3)	91
**11**	706.1125 (+3)	706.1151 (+3)	99
**12**	706.1 (+3)	706.4 (+3)	95
**13**	759.8220 (+3)	759.8236 (+3)	99

* purity for **1** was assessed by ^{1}H-NMR, while purity for **2-12** was worked out by peak integration from TIC chromatogram.

4.1 High-resolution mass spectra for compounds **2-11** and **13**

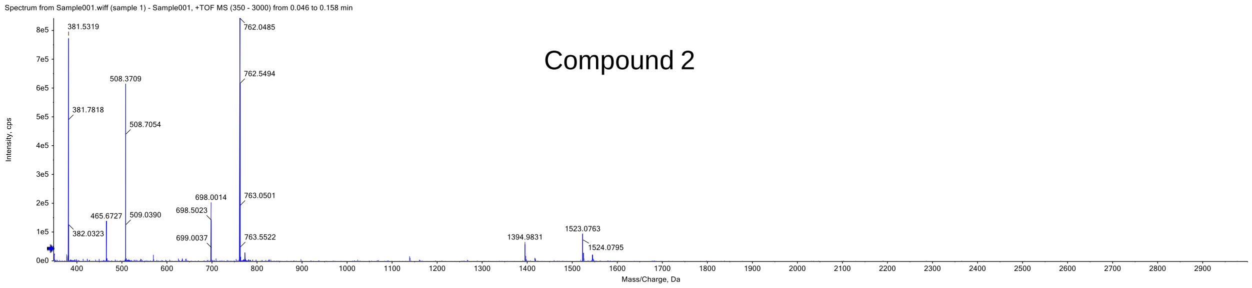

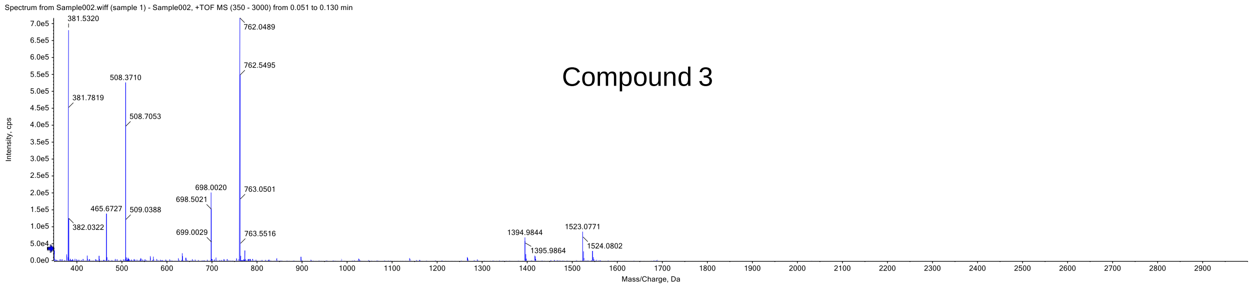

Compound 4

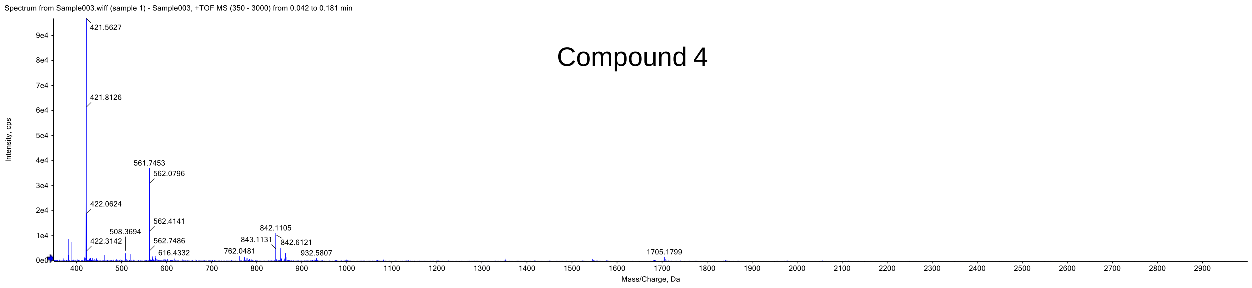

Compound 5

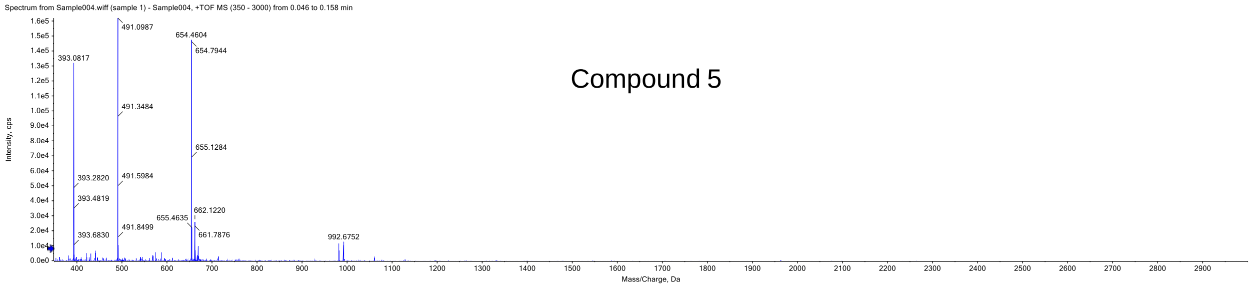

Compound 6

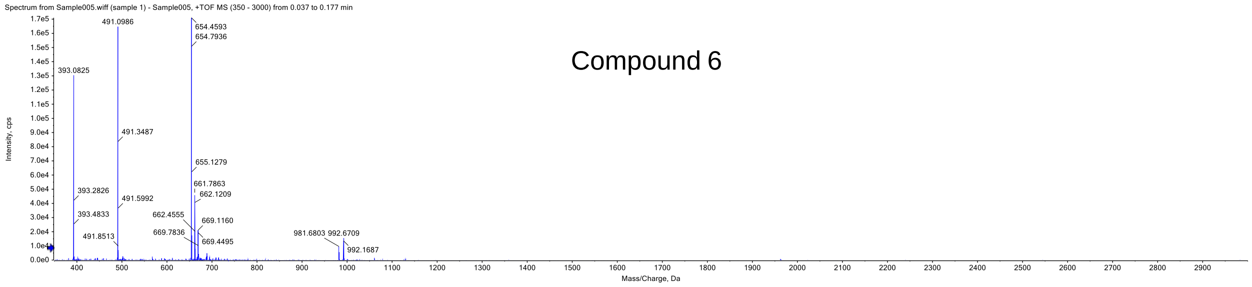

Compound 7

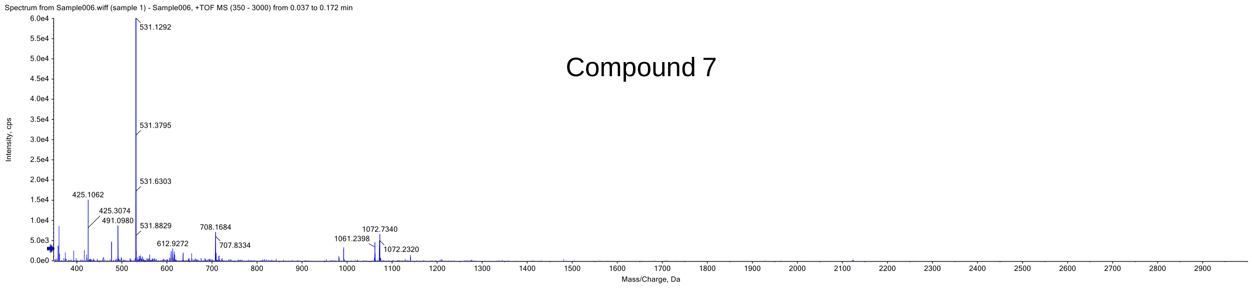

Compound 8

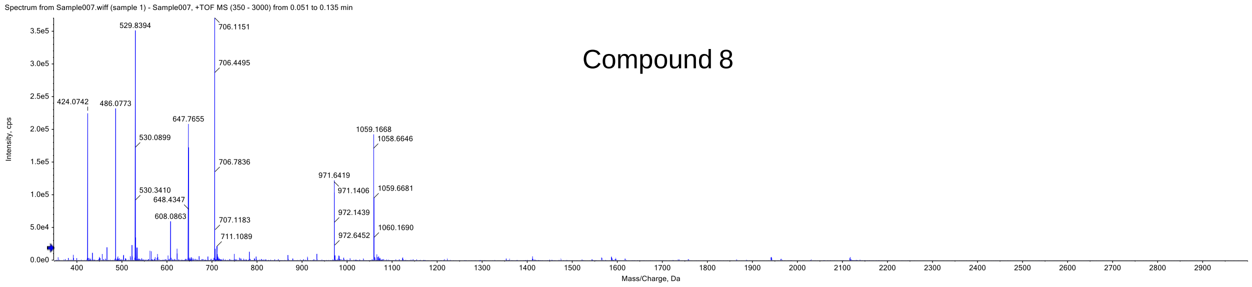

Compound 9

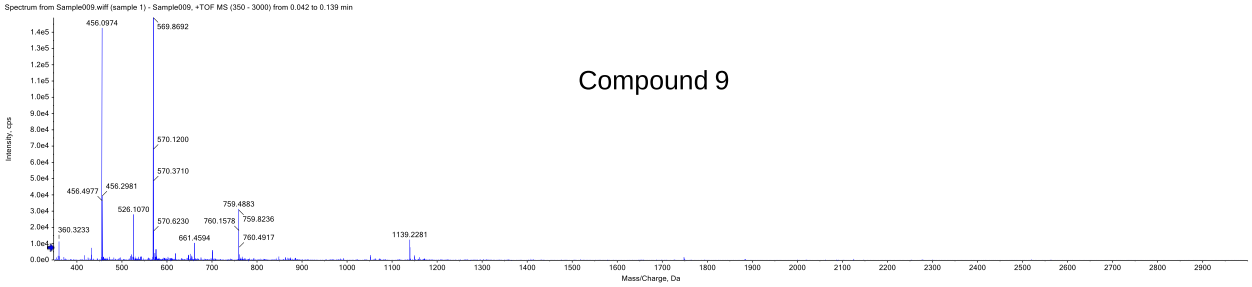

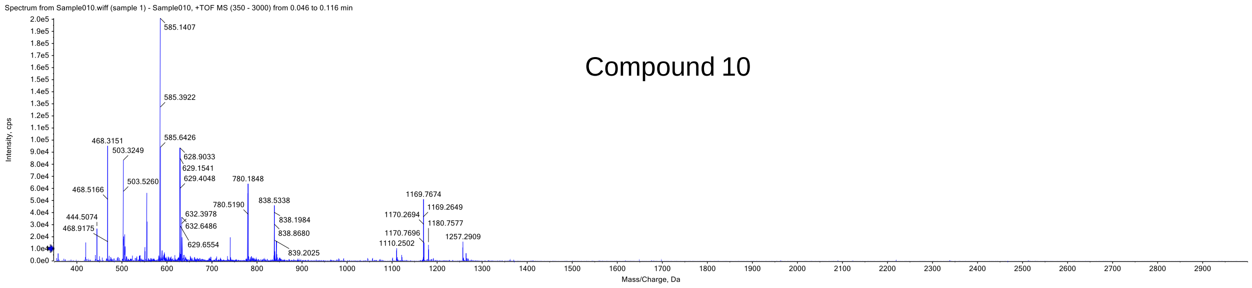

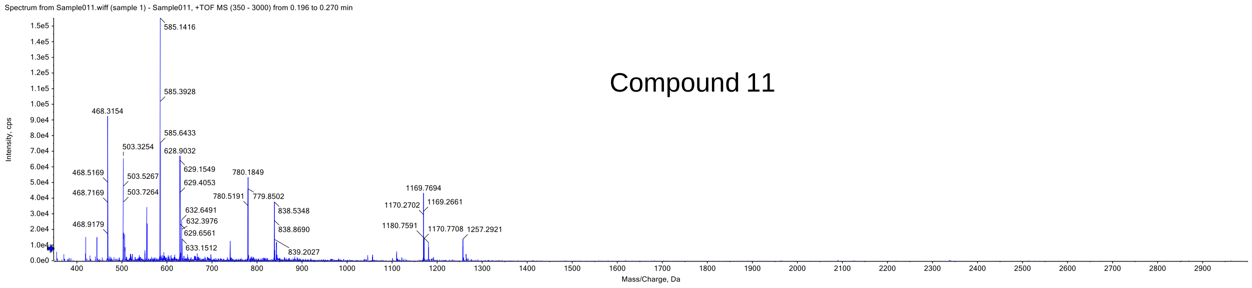

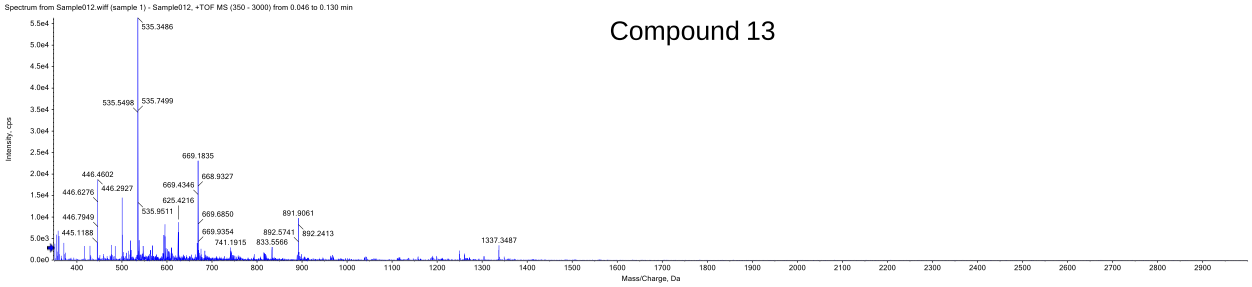

5. References

1. Kvach, M.V.; Ustinov, A.V.; Stepanova, I.A.; Malakhov, A.D.; Skorobogatyi, M.V.; Shmanai, V.V.; Korshun, V.A. A Convenient Synthesis of Cyanine Dyes: Reagents for the Labeling of Biomolecules. *European Journal of Organic Chemistry* **2008**, *2008*, 2107-2117, doi:10.1002/ejoc.200701190.
